# Electrospun Fibers as a Dressing Material for Drug and Biological Agent Delivery in Wound Healing Applications

**DOI:** 10.3390/bioengineering5010009

**Published:** 2018-01-27

**Authors:** Mulugeta Gizaw, Jeffrey Thompson, Addison Faglie, Shih-Yu Lee, Pierre Neuenschwander, Shih-Feng Chou

**Affiliations:** 1 Department of Mechanical Engineering, College of Engineering, The University of Texas at Tyler, Tyler, TX 75799, USA; mgizaw@patriots.uttyler.edu (M.G.); jthompson42@patriots.uttyler.edu (J.T.); AFaglie@patriots.uttyler.edu (A.F.); 2 School of Nursing, College of Nursing and Health Sciences, The University of Texas at Tyler, Tyler, TX 75799, USA; ShihYuLee@uttyler.edu; 3 Department of Cellular and Molecular Biology, The University of Texas Health Science Center at Tyler, Tyler, TX 75708, USA; Pierre.Neuenschwander@uthct.edu

**Keywords:** electrospun fibers, drug release, small molecule drugs, proteins and peptides, gene vectors, composites

## Abstract

Wound healing is a complex tissue regeneration process that promotes the growth of new tissue to provide the body with the necessary barrier from the outside environment. In the class of non-healing wounds, diabetic wounds, and ulcers, dressing materials that are available clinically (e.g., gels and creams) have demonstrated only a slow improvement with current available technologies. Among all available current technologies, electrospun fibers exhibit several characteristics that may provide novel replacement dressing materials for the above-mentioned wounds. Therefore, in this review, we focus on recent achievements in electrospun drug-eluting fibers for wound healing applications. In particular, we review drug release, including small molecule drugs, proteins and peptides, and gene vectors from electrospun fibers with respect to wound healing. Furthermore, we provide an overview on multifunctional dressing materials based on electrospun fibers, including those that are capable of achieving wound debridement and wound healing simultaneously as well as multi-drugs loading/types suitable for various stages of the healing process. Our review provides important and sufficient information to inform the field in development of fiber-based dressing materials for clinical treatment of non-healing wounds.

## 1. Introduction

Wound healing is a complex tissue regeneration process that the body undergoes as a response to wound openings or missing cellular structures as a result of various types of traumatic injury. To facilitate effective wound healing, a wound site is typically covered with a sterile dressing material to avoid infection and to promote the healing process. Gels and creams with a typical drug load of only 5% at the highest are typically applied to the wound site as a treatment method in the clinical setting [1]. These dressings require frequent changing and monitoring/cleaning of the wound site. In patients with non-healing wounds such as diabetic wounds and venous ulcers, the insufficient therapeutic efficacy of the creams and gels coupled with the frequent wound manipulation can often be painful for the patient and cost- and labor-intensive for the healthcare system. With improvements in advanced medical fabrics, a new generation of the wound dressing materials is expected to be able to carry a higher level of drugs and thus provide sustained release properties that will enhance the wound healing process and alleviate much of the painful repetitive procedures of frequent changes of dressing materials. In addition, these new medical fabrics can potentially be incorporated into a multifunctional wound bandage, providing treatment strategies for various types of wounds, locations of the wounds, and conditions of the wounds. The goal is to improve the quality and rate of wound healing while still being able to customize the therapeutic procedures during wound healing.

Different methods have been studied for the fabrication of polymeric fibers, such as melt blowing, phase separation, self-assembly, and temple synthesis. Among these available technologies, drug-eluting fibers made by electrospinning are potential candidates for the formulation of medical fabrics for wound healing. Electrospinning is a simple, robust, and cost-effective method to produce drug-containing fibers with diameters ranging from tens of nanometers to several micrometers [2]. The result is a layer of non-woven fiber mesh that exhibits the texture of typical textiles with a porous structure allowing drainage of the wound exudates and permeation of atmospheric oxygen to the wound. In addition to these advantages, electrospinning is compatible with the incorporation of various types of drugs and/or other drug delivery systems to facilitate generation of various composite dressing materials suitable for controlled release of biological agents at different stages of the wound healing process.

In this review, we discuss different types of wounds and the stages in the wound healing process with respect to the potential use of electrospun fibers as the dressing material in wound healing. In particular, we focus our review on the types of drugs that can be incorporated in electrospun fibers and their drug release behaviors in the context of wound healing. These drugs include small molecule drugs, growth factors, peptides, and non-viral gene vectors. In addition, we provide a brief overview of fiber-based composites that include nanoparticles and/or micelles for generation of multifunctional wound dressings. This review provides a comprehensive discussion on the current status of electrospun fibers for wound healing applications.

## 2. Wound Healing

A wound can be defined as the disruption or loss of the cellular and/or tissue structure that prevents local tissues from performing their normal biological functions [3]. Since wounds vary in sizes, shapes, and conditions, several methods are used to classify wounds (Table 1).

### 2.1. Classification of Wounds

The most common way to classify wounds is based on the nature of the wound healing process involved (i.e., acute versus chronic wounds). Specifically, acute wounds are the result of mechanical injuries from external factors resulting in tissue abrasions or tears to the skin and/or flesh. In contrast, chronic wounds are defined as occurring when the normal healing mechanisms of the body are somehow inhibited or disrupted or when tissues are constitutively exposed to damaging environmental factors [5,6]. Particularly problematic is that the healing process of chronic wounds can be readily disrupted by the necessary presence of inflammatory factors, which stimulate the immune system to recruit more macrophages and neutrophils to the wound bed. This causes the additional release of inflammatory cytokines whose presence results in mass production of metalloproteinase that can subsequently disrupt the healing cycle, all of which is characterized by a prolonged inflammatory phase [5]. This is one of the contributing factors that chronic wounds typically take longer to heal (>12 weeks) in comparison to acute wounds (8–12 weeks) that follow a more normal healing and remodeling process [7]. The most common types of chronic wounds that account for 70% of the cases are venous, pressure, or neuropathic ulcers [8]. These non-healing wounds lead to growth of bacteria and other pathogens at the wound sites that elicit increased inflammation, subsequently inhibiting the healing process and resulting in greater chances of complications.

Wounds can also be classified based on the types of closure methods used during the healing process and include Primary Intention, Secondary Intention and Tertiary Intention wound categories. Primary Intention wounds are those that can be treated by closing the surface surrounding the wound using stiches, staples, skin glue, or tape. Secondary Intention wounds are those that involve a wider area of damaged tissue that cannot be closed or stitched together. Examples of Secondary Intention wounds include leg ulcers, pressure damage wounds, and lacerations. Tertiary Intention wounds can also be referred to as delayed Primary Intention wounds and are wounds that are left open intentionally to allow for drainage to occur. These wounds are typically closed once drainage has completed satisfactorily [3,4,6].

A third wound classification method used is based on the color displayed by the damaged or injured tissue. This category includes wounds exhibiting black (necrotic tissue), green (infected tissue), yellow (sloughy tissue), red (granulating tissue), or pink (epithelial tissue) colorations. These categorizations are often used for selecting the appropriate dressing to facilitate a speedy healing process [4]. Additionally, wounds can be classified by the depth of the skin layers that have been affected. Injuries affecting only the epidermis are referred to as superficial wounds, while those affecting both the epidermis and the inner dermal layer (which contains blood vessels, sweat glands and hair follicles) are referred to as partial thickness wounds [9]. These are used largely to describe the severity of pressure sores and severe scale exits [10].

### 2.2. Wound Healing Cycles

**Figure 1:**
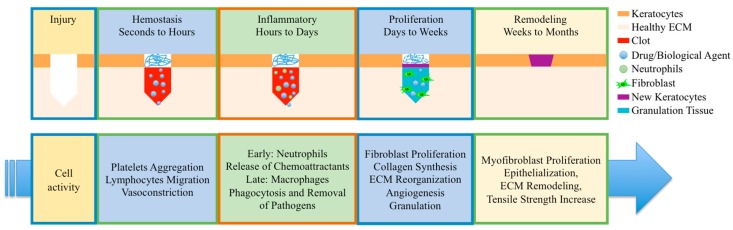
Schematics of typical wound healing cycles and the corresponding cellular activities in each stage.

Wound healing is a complex biological process that involves crosstalk between different biological systems for the proper regeneration of cells and tissues and to restore homeostasis and normal biological function [3,11,12]. The typical healing process for a wound includes four overlapping and interdependent stages: hemostasis and coagulation [13]; inflammation; proliferation; and remodeling (Figure 1) [6,14].

Hemostasis occurs immediately after tissue injury with the purpose of stopping the loss of blood (exsanguination). Tissue damage results in leakage of blood out of the vessels into the adventitia, where high levels of Tissue Factor protein are present. The contact of plasma factors with Tissue Factor triggers the blood coagulation cascade in the area of damage to generate the fibrin clot. In conjunction with this activity, blood platelets that come into contact with extracellular matrix collagen are activated to form an interim platelet plug that seals the breach until the fibrin clot can form and consolidate the wound. Protein and peptide byproducts of the enzymatic reactions involved in coagulation serve as signals to surrounding cells to recruit immune cells to the clot, which is ultimately composed of fibrin molecules, fibronectin, vitronectin, and thrombospondins [3,6,15]. Although initially involved in hemostasis, the clot subsequently serves as a matrix for immune cells in the subsequent stages of tissue repair and wound healing [14,16].

The inflammatory stage is activated during the hemostasis and coagulation phase and can last up to three days [9,17,18]. Recruiting of neutrophils is triggered at early stages due to the presence of degranulated platelets and byproducts of bacterial degranulation followed by the appearance and transformation of monocytes at later stages [19]. Neutrophils help in the early phase of the inflammatory stage in cleaning bacteria by engulfing them and degrading necrotic tissue. Macrophage enters the injured site within three days and promotes phagocytosis of pathogens and cell debris. They also up-regulate the secretion of growth factors, chemokines, cytokines, and activating the next phase of the wound healing process. Macrophages provide benefits in the wound healing process by boosting defense, promoting and resolving inflammation, removing apoptotic cells, and supporting cell proliferation and tissue restoration following injury [3,6].

The proliferative stage lasts from the third day till the 24th day. When current injury has stopped, hemostasis has been attained and an immune response effectively set in place, the acute wound moves toward tissue repair [18]. The fibrin/fibronectin matrix is replaced with newly formed granulation tissue [14]. At this stage the tissue is very delicate and does not have the normal organization of the surrounding tissues. At the macroscopic level, this phase of wound healing can be perceived as an ample construction of granulation tissue. A fibrous network is formed by the multiplication of fibroblasts forming collagen fibrils. Inflammation also starts to subside at this stage, but in open abnormal wounds the proliferative phase can be prolonged due to more fiber networks needing to be formed [3,6,20].

The remodeling stage occurs from the 24th day and extends to one year [18]. As the concluding phase of wound healing, the remodeling phase is accountable for the growth of new epithelium and final scar tissue formation by achieving a balance between synthesis, deposition, and degradation of the tissues [14]. The wound contracts and becomes smaller due to being pulled by collagen fibers in response to the wound being filled by granulation tissue. While the preliminary deposition of collagen bundles is vastly disorganized, the new collagen matrix becomes more oriented and cross-linked over the phase [3,6]. The healed tissue archives 80% of original tensile strength since some cellular components and their organization cannot be fully recovered during healing [16,17].

### 2.3. Non-Healing Wounds

A widely accepted mechanism in forming chronic wounds is the interruption or deregulation of one or more of the wound healing cycles that lead to development of non-healing chronic wounds from acute wounds [17]. Furthermore, chronic wounds show improper deregulation of protease and their inhibitors [21]. Matrix components, including fibronectin, as well as various key growth factors are degraded by serine proteases. Non-healing wounds have also been observed with a depletion or small number of chemokine that are needed to recruit bone marrow and endothelial progenitors to the site of injury [22]. The presence and growth of blood vessels surrounding the injured tissue are paramount to the wound healing process, since they supply nutrition and oxygen. Angiogenesis and vasculogenesis contribute to the formation of blood vessels during injury tissue repair [23]. A shortage of any of these factors will lead to the development of chronic wounds.

Management of chronic wounds includes the application of an antimicrobial dressing that help in reducing inflammation and regulation of other pathogens [20]. Wound dressings have been used since ancient times to stop bleeding, and they consist of honey, animal oils or fat, cobwebs, mud, leaves, sphagnum moss, and animal dung. Current commonly used dressing is cotton gauze, and some of its shortcomings include damaging newly formed tissue during removal and causing rapid dehydration of the wound bed, leakage of exudate the might result in infection, and reaction of cotton fibers with foreign body [24]. Ideal wound dressings must meet one or several of the following functions: (1) spot bleeding and protect wound from pathogens, (2) restoration of normal bacterial balance in wound, (3) reduction of inflammation due to unregulated matrix metalloproteinase, and (4) a suitable environment for the control of odor and promotion of autolysis [24]. Based on the types of treatment, developments of wound dressings are commonly categorized in to four groups: passive, interactive, advanced, and bioactive wound dressing. Passive dressings are used to protect mechanical trauma and limit the entrance of pathogens to the wound. Interactive dressings are made from polymeric films, and they facilitate the flow of moisture and air from the environment while providing a barrier from bacteria or other environmental contaminants. Advanced dressings are able to provide and retain a moist environment for the wound and facilitate the healing process. Bioactive dressing works by including drug delivery systems and/or biological agents to stimulate cellular responses in the healing process. The advance of materials science and biomedical engineering has enabled the development of bioactive dressings using natural/synthetic polymers in the form of fibers as a carrier to deliver drugs and biological agents. Fiber based dressing are advantageous to traditional dressing since they provide wide range of advantages including creation of moist and warm environment, remove excess exudates, allow gaseous exchange, and do not release fiber materials to the wound hence minimizing risk of infection [25]. One of the most popular and promising methods in preparation of fibers is electrospinning.

## 3. Electrospun Fibers

Fibers obtained from electrospinning have gained popularity in the field of drug delivery and are considered as ideal dressing materials for non-healing wounds since the method is versatile and can deliver various biological agents long-term to local tissues at the wound site [26,27,28]. Not only do they provide physical protection to the wound, but they also have the capacity to be incorporated with a high amount of drugs (up to 40% loading), where the release of which can be adjusted by changing the types and compositions of the materials in the fibers [29]. A large variety of materials can be used to produce electrospun fibers in the pursuit of medical fabrics in wound dressing [26], and these materials can be categorized into natural and synthetic polymers [27,30]. In addition, hydrophilic polymers are ideal for encapsulation of small molecules, proteins, peptides, and gene vectors. The release rate of hydrophilic systems is fast and hence limiting its long-term applications. In contrast, the use of hydrophobic polymers is ideal for controlled release purpose. However, the process requires the use of harmful organic solvents that might affect the stability of the biological agents resulting in the decrease of pharmacological efficacy. In this section, we summarize the most common polymers employed in electrospinning process for wound dressing materials.

### 3.1. Natural Polymers

Natural polymers have several benefits including being fairly abundant and accessible, as well as being biocompatible, biodegradable, and nontoxic in most cases [31]. In addition, their structural similarity to the ECM promotes and stimulates wound healing process. Others have shown the benefits of using natural polymers for the repair of damaged tissues and consequently in skin regeneration [32]. However, natural polymers are sometimes extremely difficult to electrospin alone due to their molecular structure. This issue can be overcome with the introduction of a synthetic polymer as a carrier to pair with the natural polymer for electrospinning (Table 2). For example, chitosan, a natural occurring polysaccharide, has become a fairly popular material for wound dressing since it is hemostatic, antimicrobial, nontoxic/biocompatible and biodegradable, and capable of sustaining drug release to facilitate wound healing [33,34]. Chitosan is insoluble in water, so the use of organic solvents is necessary during electrospinning. It also exhibits high viscosity at low concentrations when dissolved in organic solvents, which makes electrospinning very difficult. Pakravan et al. showed how these problems can be overcome in electrospinning by paring chitosan with polyethylene oxide (PEO) using 4wt.% stock solution of each at various ratios of 50/50, 70/30, 80/20, and 90/10 chitosan/PEO blends [35]. The paring of these two polymers improved the electrospinnability through strong hydrogen bonding between chitosan and PEO chains. Other popular natural polymers commonly used in blends for electrospinning in application of wound healing include alginate [36], gelatin [37,38], cellulose [39], collagen [40,41], hyaluronic acid [42,43,44], keratin [45], silk fibroin [46], and zein [47,48].

### 3.2. Synthetic Polymers

Synthetic polymers that are biodegradable and biocompatible have been widely used in electrospinning for wound healing purposes. They can be blended with other synthetic or natural polymers to provide sustained release of drugs [2,61]. These polymers can be separated into water soluble and insoluble polymers (Table 2). This characteristic strongly affects the ability of polymers to degrade over time and therefore determine the mechanism in drug release for wound healing applications.

Poly(*ε*-caprolactone) (PCL) belongs to aliphatic polyester that is extremely popular in the biomedical research sector due to its ease in processing of biomaterials. In addition, its properties, such as being nontoxic, biodegradable, biocompatible to many drugs, and easily accessible, have lent it to be a prime candidate as a long-term drug-delivery carrier. PCL is highly hydrophobic and degrades over several months, and therefore, it is possible to adjust its degradability by blending with a hydrophilic polymer. Ponjavic et al. demonstrated blending water soluble PEO to PCL and showed that the surface properties were greatly improved due to the hydrophilic nature of PEO [62]. Studies have also been done to determine the effect of hydrophilic drugs on the fiber formation and release profile of PCL fibers. For example, Luong-Van et al. showed that increasing loading of the hydrophilic heparin resulted in the decrease of fiber diameter and a sustained release behavior for up to 14 days [63]. Similar to PCL, PLGA is another synthetic polyester that is biodegradable and biocompatible, where its strong mechanical properties make it an ideal candidate as a drug release vehicle. PLGA and PCL exhibit prolonged degradation times, where t_1/2_ = 30 days for PLGA and t_1/2_ >18 months for PCL [64,65,66]. Ranjbar-Mohammadi et al. explored the potential of tuning of hydrophilic tetracycline hydrochloride from blend and core shell fibers using PLGA and gum tragacanth [55]. Results suggested that blended fibers exhibited a much higher cumulative release and initial burst of tetracycline hydrochloride than pure PLGA fibers over 75 days, whereas the core shell structure displayed an intermediate cumulative release profile between blend fibers and pure PLGA fibers. PU and PVDF are synthetic water insoluble polymers that exhibit similar hydrophobic characteristics as PCL and PLGA with various degradation rates that range from several weeks to months [67,68,69].

In contrast to water insoluble polymers, poly(vinyl alcohol) (PVA) is a synthetic polymer that is soluble in water, biocompatible, and nontoxic. PVA fibers rapidly disintegrate in aqueous solutions resulting in the fast release of drug due to dissolution of the carrier materials [70]. PVA is quite compatible with chitosan as suggested by a study using blend fibers of PVA/chitosan for dressing materials on diabetic wounds [28]. Results showed that the rats treated with PVA/chitosan fiber dressings had an increased wound healing rate as compared to those untreated rats. Similar to PVA, the hydrophilic nature of PEO causes it to disintegrate faster than the hydrophobic polymers. Kim et al. showed the ability to tune release characteristics of protein lysozyme through blending hydrophobic polymers (PCL, PLLA, and PLGA) with hydrophilic PEO [71]. In vitro drug release showed that the PCL/PEO blend received the smallest burst and the most prolonged release profile. Polyethylene glycol (PEG) is also a hydrophilic polymer that has been studied for drug release using blends with a hydrophobic counterpart. Studies have shown that PEG can aid in the healing process of in vivo wounds. Bui et al. explored the effects of curcumin loaded PCL/PEG fibers on wound closure rates in rats, and found that the curcumin loaded blended fibers achieved a 99% wound closure as compared to 90% with just curcumin loaded PCL fibers at 10 days [72]. By controlling the hydrophobicity of these synthetic polymers, a blend polymeric fiber platform is ideal for controlled release of drugs in wound healing applications [60,73,74].

### 3.3. Electrospinning Parameters

There are several parameters in electrospinning that must be determined in order to produce homogeneous and uniform fibers. These parameters, including solution properties, applied voltage, distance from the tip of the needle to the collector plate, and feeding rate of the polymeric solution, vary with the types of polymers and solvents used during the process. Solvents with moderate boiling points are usually ideal as they are volatile enough to evaporate between the needle tip and collector plate without evaporating too fast and clogging the needle tip [75]. The solvent is also very important when determining the drug that will be incorporated for wound healing purposes. The drug must be able to dissolve in the solvent for complete encapsulation in the fibers. The parameter that dictates the determination of subsequent parameters is the polymer solution viscosity. Megelski et al. showed the influence of solution viscosity on the formation of polystyrene (PS) fibers as fiber diameter increased with increasing solution viscosity [76]. The next parameter that affects electrospun fibers is the applied voltage, which varies with types of polymer solutions used. Each polymer has a critical voltage, or the electric field, that will produce fibers as studies showed that using applied voltage past the critical voltage while holding all other parameters steady created beads during electrospinning of PVA [77]. The solution flow rate has a critical value to produce smooth fibers, and is closely related to the size of the Taylor cone when combined with applied voltage. The increase in flow rate increased fiber diameter and allowed less time for the evaporation of solvent during fiber formation, which can cause beading [76]. The distance from the tip of the needle to the collector has shown an effect on the creation of fibers during electrospinning. Matabola and Moutloali varied the distance when electrospinning poly(vinyledene fluoride) (PVDF) solutions, and found that fiber diameter decreased when increasing the distance, suggesting a complete evaporation of the solvent [78]. While each of these parameters is considered to have its own unique effect on fiber formation during electrospinning, usually these parameters are adjusted together to find the most efficient electrospinning condition. Table 2 provides commonly used natural and synthetic polymers and their electrospinning parameters.

Producing fibers with good structure are important for prolonged delivery of wound healing drugs. Another factor that affects the drug release in wound healing is the type of encapsulation technique used when creating the fibers. There are two main types of methods to create drug-loaded fibers (i.e., uniaxial blends and coaxial core-shell electrospinning). When creating blend fibers, the drug is dissolved in the blend polymer solution and allowed for mixing. For this method, the choice of solvent is important since the particular solvent must be able to dissolve polymers and drugs to create fibers. Uniaxial blends allow the use of hydrophilic and hydrophobic polymers and drugs, if miscible; however, drug partitioning in hydrophilic/hydrophobic phase of the fibers may lead to unexpected release characteristics. Usually this means the drug and polymer must have the same hydrophobicity. Coaxial core-shell electrospinning is a method that allows the formation of layered structure in the radial direction of the fibers. It accommodates much more polymer combinations as compared to the blend fibers since the core and the shell can be from different polymer-solvent system. Core-shell fibers provide the ability to intentionally incorporate hydrophilic and/or hydrophobic drugs at the core of the fibers while the shell serves as a protective layer to prevent burst of the surface drugs [30]. This method not only allows more possible formations of drugs and polymers, but also can prolong the release of the drug to the wound site. One of the drawbacks in core-shell fibers is the swelling of the core polymer, which can cause rupture of the shell layer and expose the drug-containing core to outside environment [79].

## 4. Release of Small Molecule Drugs

Small molecules represent the majority of the drugs used in treatment of non-healing wounds. A wide variety of small molecule drugs have been incorporated into electrospun fibers for various drug release applications [80]. In this section, we review some of the popular hydrophilic and hydrophobic small molecule drugs incorporated in electrospun fibers and their release behaviors for the potential application in wound healing.

### 4.1. Hydrophilic Drugs

The use of hydrophilic small molecule drugs to achieve sustained release can be a challenge due to their high solubility in physiological solutions and compatibility with polymers during electrospinning leading to poor or preferential partitioning in the polymer matrix. In addition, the compatibility of the drug in the polymer matrix is indicative of encapsulation efficiency and ability to achieve sustained release behavior. For example, hydrophilic small molecule drugs receive low solubility with nonpolar solvent-polymer systems, and therefore, drugs are likely to partition at the fiber surface, which contributes to burst release. Nonetheless, several hydrophilic drugs for wound healing applications have been incorporated in electrospun fibers and extensively studied for their release characteristics (Table 3). The physicochemical properties of small molecule drugs (i.e., aqueous solubility and Log P) and their loading in the polymer matrix are important factors to consider for drug release behaviors. For example, ciprofloxacin, a hydrophilic antibiotic drug for wound healing, showed burst release behavior in 2 min when incorporated into a water-soluble polymer [81] whereas sustained release of the same drug was reported up to 10 days (80% cumulative) when using a hydrophobic polymer [82]. Similar release behaviors with respect to the polymer used for small molecule hydrophilic antibiotics such as ampicillin [83,84,85], metronidazole [86,87,88], and cefazolin [89,90] were reported by others. In addition, drug loading in the polymer matrix plays an important role in the release mechanism of small molecule drugs from fibers. Current trends to achieve sustained release form uniaxial fibers have been utilizing hydrophobic polymers to provide large surface tension to the release media leading to slow release. However, by increasing the loading of hydrophilic drug in the fibers, the overall hydrophobicity changes allowing a better penetration of the release media within fiber mesh. In addition, high drug loading increases surface drug content in fibers, which promotes the burst release behavior. Therefore, a better understanding of the physicochemical properties of hydrophilic small molecule drugs, such as their aqueous solubility and LogP (Table 3), in addition to the polymer used for electrospinning may benefit the development of sustained release wound dressing materials from electrospun fibers.

### 4.2. Hydrophobic Drugs

Hydrophobic drugs are generally able to provide a sustained release profile for an extended period of time as comparing to hydrophilic drugs due to their poor solubility in physiological conditions and preferred partitioning in the insoluble polymer matrix than diffusion into the release media (Table 3). In this section, we provide information on the most widely used hydrophobic small molecule drugs in wound healing as well as the commonly used polymeric fibers to achieve the sustained release behavior. One of the most widely studied topical wound healing medications is phenytoin where its mechanism of action includes up-regulating collagen deposition that promotes the formation of fibroblasts, granulation, and other connective tissues with the ability to down-regulate collagenase activity, bacterial colonization, and the formation of wound exudate [104,107,108]. In particular, Zahedi et al. demonstrated the encapsulation of phenytoin in PVA (20% *w*/*w*) and PCL (17.4% *w*/*w*) electrospun fibers [104]. In vitro release of phenytoin suggested a cumulative release of 90% and 15% in PVA and PCL fibers, respectively. The differences in release profiles were attributed to the hydrophobicity of PVA and PCL polymer. In addition, in vivo wound closure study showed a 50% reduction of wound area after 6 days using phenytoin incorporated PVA fibers as compared to control phenytoin containing ointment. Another hydrophobic drug that has been extensively studied for wound healing is nifedipine, which serves as calcium antagonist to facilitate blood flow to the wound [109]. Using a water-soluble polymer (i.e., Eudragit), cumulative release of nifedipine in vitro showed 40% and 70% release at 1 h and 8 h, respectively [101]. In another study, nifedipine was encapsulated with PCL-based polyurethane fibers at loadings of 2.7% and 4.2% [102]. In vitro drug concentration in the release media showed positive correlations with drug loading; however, no significant change was found in cumulative drug release with 50% nifedipine release at 24 h followed by 70% release at the end of the study (i.e., 72 h). The slow release behaviors were also observed for curcumin [95,96], ketoprofen [97,98,99,100], vancomycin [105], and methylene blue [106] providing the use of proper insoluble drug carriers.

In general, drug types and loading combined with the use of soluble and/or insoluble polymer matrix in electrospinning play a significant role in the release behavior of small molecule drugs. Given that the development in advanced dressing materials using electrospun medical fabric often requires the use of multiple drugs at different stage of the healing process, a better understanding of drug–polymer interactions will benefit the design process of the fiber-based dressing materials.

## 5. Release of Macromolecules

Wound healing involves complex cellular activities controlled by signaling networks from various growth factors, cytokines, and chemokines [110]. The delivery of macromolecules to the wound site becomes of particular interests for non-healing wounds since it provides a method to remodel normal wound healing cycles. In this section, we review the release of macromolecules (i.e., growth factors and peptides) using electrospun fibers for wound healing.

### 5.1. Growth Factors

**Figure 2:**
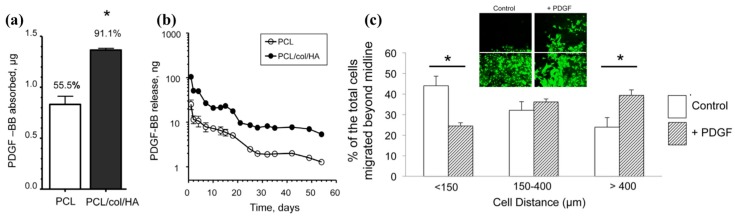
(**a**) PDGF-BB (1.5 μg) was passively absorbed by PCL and PCL/col/HA fibers at 4 °C for 24 h using a PBS bath (300 μL); (**b**) In vitro release profiles of PDGF-BB from fibers over 56 days; (**c**) Release of PDGF-BB promoted MSCs migration using a stringent migration assay (inset shows fluorescent image of the MSC migration) [113]. An asterisk denotes *p* < 0.01. Figures were obtained from an open access article distributed under the Creative Commons Attribution License.

Growth factors are cellular protein secretions that are crucial for tissue remodeling due to their influences on cell cycles and cell fate. Methods to incorporate growth factors in/on electrospun fibers include blend polymers, emulsion, coaxial fibers, and immobilization. The release rates are dictated by the types of technique used. For example, a fast release (>90%) of epidermal growth factor (EGF) and basic fibroblast growth factor (bFGF) were observed in 5 days and 17 days, respectively, whereas a sustained release of platelet-derived growth factor (PDGF-BB) and vascular endothelial growth factor (VEGF) over 1 month was found using blend fibers made from dual electrospinning of collagen and hyaluronic acid [111]. The sustained release is due to the encapsulation of the growth factors in gelatin nanoparticles within the fibers. Others demonstrated the release of bFGF and EGF from coaxial fibers made from block copolymer of PCL-PEG where bFGF solution was encapsulated in the core surrounded by EGF immobilized PCL-PEG shell [112]. The conjugation of EGF to PCL-PEG shell showed a slow release of 2% in a week whereas 30% of bFGF was released in 12 h due to diffusion. In another study, PDGF-BB was absorbed into PCL/collagen/nanohydroxyapatite blend fibers and pure PCL fibers where the blend fibers showed a higher absorption of the growth factor followed by a higher concentration of the PDGF-BB in the release media from the blend fibers as compared to those from pure PCL fibers over 8 weeks of investigation (Figure 2) [113]. Others incorporated transforming growth factor (TGF-β3) into hyaluronic acid (HA) fibers and PCL fibers, and in vitro release study suggested 78% of TGF-β3 was released from HA fibers as compared to 18% from PCL fibers at 2 days followed by 95% release from both fibers at 21 days [114]. Furthermore, others have shown effectiveness of fibroblast growth factor (FGF2) [115], insulin-like growth factor (IGF) [116], granulocyte macrophage colony stimulating factor (GM-CSF) [117], and connective tissue growth factor (CTGF) [118] in promoting cell proliferation, which benefits wound healing (Table 4). In general, with proper incorporation techniques of the growth factors in electrospun fibers, sustained release of the macromolecule agents can be achieved for days to months, which is beneficial for non-healing wounds.

### 5.2. Peptides

Peptides, such as human cathelicidin peptide LL37, have shown their ability in controlling wound infections through the antibiotic efficacy. In a study, an antimicrobial peptide motif (Cys-KR12) originated from LL37 was immobilized onto silk fibroin fibers [129]. Results showed immobilization processes at various Cys-KR12 concentrations achieved more than 90% yield, and Cysk-KR12 immobilized fibers were able to maintain antibacterial properties for 3 weeks. Furthermore, the study suggested the important role of Cysk-KR12 in wound healing by activating biological activities of keratinocytes, fibroblasts, and monocytes. In another study, a proline-rich peptide (Chex1-Arg20) was electrospun with PVA into fibers for treatment of *Acinetobacter baumannii* infected wounds in mice [130]. Results showed a significant decrease in wound size after 3 days when using Chex1-Arg20 incorporated PVA fibers, whereas the antimicrobial activity of the peptide-loaded fibers was significantly improved. Similarly, Lee et al. incorporated bone forming peptide1 (BFP1) into electrospun PLGA fibers coated in polydopamine (PD) for use of bone regeneration in vivo. Results showed increased bone growth in mice treated with PLGA, PLGA/DP, and PLGA/DP/BFP1 with PLGA/DP/BFP1 having the greatest increase in bone growth [131]. Shao et al. conjugated peptide sequence E7 on electrospun PCL fibers and studied the effects of E7 on the formation of mesenchymal stem cells (MSCs) [132]. After implantation of the E7/PCL fiber meshes into cartilage defective rat knees for 7 days, immunofluorescent staining suggested that the cell growth on the PCL/E7 fibers had a higher percentage of MSC surface markers than the Arg-Gly-Asp peptide (RGD) control group. They also found that the PCL/E7 fibers absorbed less inflammatory cells than the PCL/RGD fibers.

## 6. Release of Gene Vectors

Gene therapy, as its name implies, is a medical approach that utilizes the delivery of genes to the target cells and/or the use of biological agents such as growth factors to trigger genetic events to further modulate cell behaviors. Similar to other disease states, the success of gene therapy in wound healing is closely associated with the development of delivery systems for gene vectors, which will determine the encapsulation efficiency and release characteristic of the gene. Specifically, therapeutic efficacy and pharmacological results in wound healing depends on gene release rate, which is mediated by cellular uptake during endocytosis followed by biological events of transcription and translation in target cells resulting in the production of proteins. In this section, we review current successes in gene delivery using electrospun fibers for wound healing.

### 6.1. Non-Viral Genes Vectors

**Figure 3:**
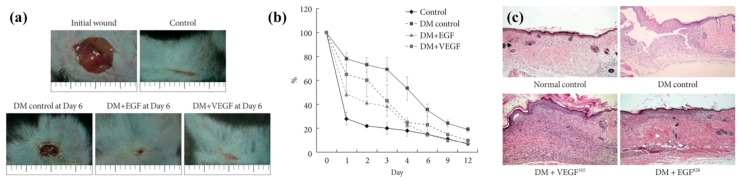
(**a**) Wound healing of a diabetic mouse (DM) model for comparison of effects on delivery of EGF cDNA and VEGF cDNA after 6 days; (**b**) Percent wound closure after receiving gene therapy from the mouse model; (**c**) Histology of the wound tissues from the animal model where tissues receiving EGF and VEGF showed restoration of the tissue structure [135]. Figures were obtained from an open access article distributed under the Creative Commons Attribution License.

Even though viral vectors (i.e., retrovirus and adenoviruses) possess a higher effectiveness and a better efficacy as compared to non-viral vectors in gene therapy, the use of viral vectors provides a greater chance to trigger immune response [133]. As a result of regulatory, non-viral gene delivery remains the primary method in gene therapy of non-healing wounds. For example, a recent clinical study (NCT01657045) was conducted using a non-viral gene vector (i.e., stromal cell-derived factor-1: SDF-1) for sternal wound edges after open heart surgery, and the results showed significance decreases in scar width (placebo: 35.9 mm and SDF-1: 18.5 mm) and defect volume (placebo: 13.9 mL and SDF-1: 1.4 mL) after 6 months of follow-up on 26 patients [134]. In addition, others observed an increase in diabetic skin wound healing rate after 12 days of follow-up using a mouse model on the delivery of minicircle-VEGF (20 μL) and pβ-EGF (20 μL) cDNA, suggesting that gene therapy can improve wound healing processes (Figure 3) [135]. In parallel to this study, histology observation on the skin tissue at caudal zone of the mice dorsal showed an increase thickness of epithelial tissue after topical administration of keratinocyte growth factor-1 (KGF-1) DNA (control: 16 ± 4 and KGF-1: 26 ± 2 μm) after 48 h while dermal thickness increased in the KGF group (255 ± 36 μm) as compared to the control group with transfected skin (162 ± 16 μm) after 120 h of follow-up [136]. Overall, these examples show that the delivery of non-viral gene vectors is a promising treatment strategy for non-healing wounds.

### 6.2. Non-Viral Genes Vectors Delivered by Fiber Platform

The incorporation of gene vectors in electrospun fibers includes encapsulation of the vectors during electrospinning process and immobilization of the vectors after the formation of the fibers. Lee et al. reviewed various gene vectors that were encapsulated in or immobilized on fibers and their release characteristics [137]. In addition, plasmid DNA was attached to linear poly(ethylene imine) segments that were further immobilized with MMP-cleavable peptides onto the amine groups of PCL-PEG blend fibers [138]. Results from the in vitro release study suggested a strong dependence of release characteristics on DNA loading in the fibers. At a plasmid DNA loading of 6.4 ± 0.5 μg per 4 cm^2^ of fibers, the release curve showed an initial burst of 60% at 12 h followed by 82% release of plasmid DNA at 72 h of incubation time in the presence of MMP-2. Using the same immobilization technique, the researchers attached MMP-responsive siRNA onto PCL-PEG fibers for treatment of diabetic ulcers [139]. The incorporation efficiency was around 77% and the release of siRNA ranged from 30% to 46% depending on the amount of siRNA incorporated onto the fibers at 72 h in the presence of MMP-2. Wound recovery from diabetic ulcer using an in vivo mouse model suggested a 65% recover rate at 7 days when using siRNA immobilized PCL-PEG fibers. Similar to these works, the immobilization of plasmid human epidermal growth factor (phEGF) onto PCL-PEG fibers showed a 2-fold increase in wound recovery rate at 7 days when comparing to the control (no treatment) on an in vivo animal model with diabetic ulcer [140]. The amount of phEGF in the re-epithelized tissue at 14 days was 11 pg/cm^2^ when using fibers as compared to 6 pg/cm^2^ from the phEGF solution. These examples show that gene delivery from fibers can promote the reconstruction of the tissue in wound and therefore improve the healing process.

**Figure 4:**
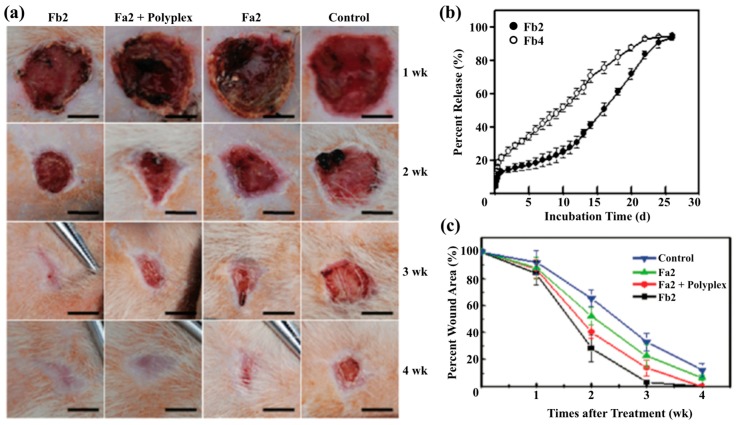
(**a**) Diabetic skin wound using a rat model for comparison of control and those subjected to delivery of pbFGF polyplexes from electrospun poly(ethylene imine)/PEG (2 kDa) core/shell fibers (Fa2: blank fibers and Fb2: fibers with pbFGF polyplexes in the core); (**b**) In vitro release profiles of pbFGF from electrospun poly(ethylene imine)/PEG core/shell fibers (Fb2: 2 kDa PEG and Fb4: 4 kDa PEG); (**c**) Percentage of wound area from the diabetic rat model [143]. Reprinted with permission from American Chemical Society. Copyright (2017) American Chemical Society.

The incorporation of plasmid DNA in electrospun fibers is challenging due to the harsh chemicals used for insoluable polymers, which have the potential to provide diffusion-based release mechanism rather than dissolution. Luu et al. demonstrated the ability to electrospin PLGA copolymer and PLA-PEG block copolymer using *N*,*N*-dimethyl formamide as the solvent for electrospinning where pCMVβ plasmid was added to the solution [141]. Results suggested a burst release of DNA at around 18% and 36% of cumulative release, depending on block copolymer content, in the first 15 min, perhaps due to the presence of DNA on the surface of the fibers instead of encapsulated inside the fibers followed by 68% to 80% of cumulative release of pCMVβ plasmid at 20 days. Others used a layer-by-layer technique to encapsulate plasmids encoding keratinocyte growth factor (KGF) in blends of PCL and PLA fibers [127]. In vitro release showed an initial 14% burst release of KGF followed by 16% release at 7 days from the fibers. In addition, core-shell fibers show a promising potential to encapsulate DNA inside the fiber structure. In a study, plasmid DNA was encapsulated in the core with the shell composed of poly(ethylenimine)-hyaluronic acid (PEI-HA) at various core-shell compositions, and the results suggested a sustained release behavior up to 60 days [142]. Furthermore, studies showed that electrospinning polyplexes of basic fibroblast growth factor-encoding plasmid (pbFGF) with poly(ethylene imine) in the core and poly(ethylene glycol) as the shell provided a sustained release up to 25 days with 12–19% of initial burst at 12 h (Figure 4) [143,144]. Overall, electrospun fibers demonstrate the potential to encapsulate DNA in the fibers and the sustained release of DNA can provide a much improved therapeutic efficacy in non-healing wounds.

## 7. Fiber Composites

Nanoparticles and micelles have been widely used in wound healing due to the ability to achieve controlled drug release. In addition, they can be easily incorporated into the traditional cream and gel formulation. In this section, we review the incorporation of nanoparticles and micelles into electrospun fibers for the use of multifunctional wound dressing materials.

### 7.1. Fiber-Micelle Composites

**Figure 5:**
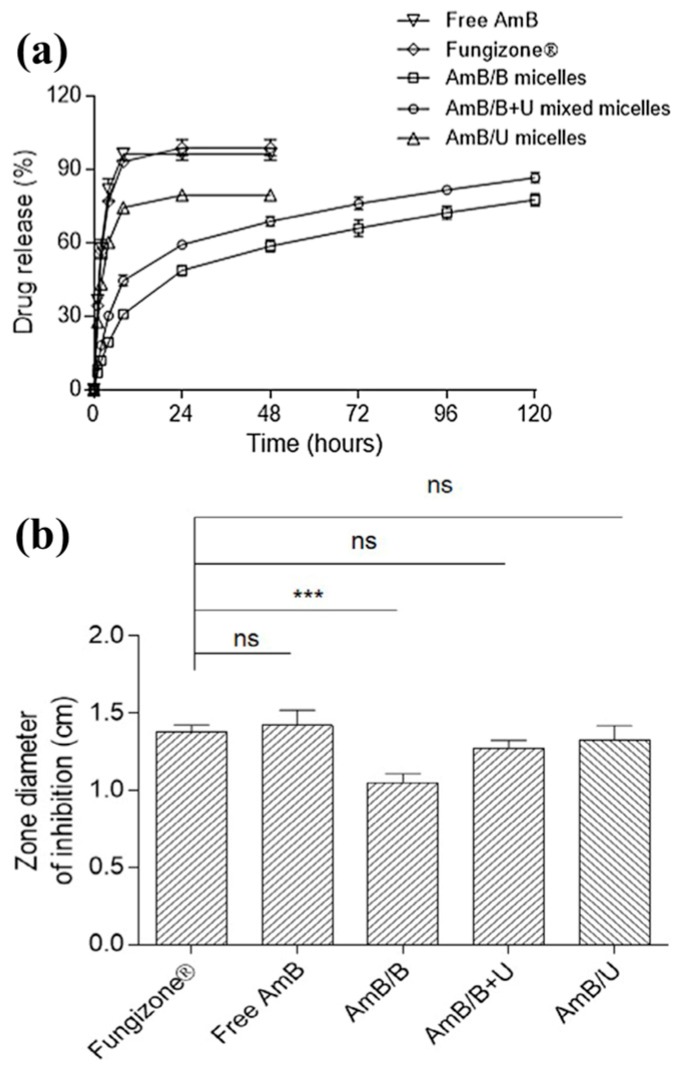
(**a**) Amphotericin B release profiles from micelles of phenylboronic acid-functionalized polycarbonate/PEG (denotes as B) and urea-functionalized polycarbonate/PEG (denotes as U) in comparison of free drug and Fungizone^®^ using dialysis; (**b**) Comparison of zone inhibition from *C. albicans* growth after applying amphotericin B containing micelles [149]. “***” denotes *p* < 0.001 and “ns” denotes no significant difference. Figures were obtained from an open access article distributed under the Creative Commons Attribution License.

Micelles have a structure of hydrophobic core surrounded by hydrophilic segments making them an ideal candidate to encapsulate drugs and release them upon contact with physiological fluids. This core shell structure creates a barrier that protects the drug which in turn allows the drug to circulate longer for a prolonged release [145,146]. In work by Redhead et al., Poloxamer 407 and 908 coatings were used with PLGA nanoparticles loaded with Rose Bengal to show the protective effect that polymeric micelles provide [147]. In vivo studies using rats showed that 30% of the dye was still present in the bloodstream 1 h after injection when loaded into the poloxamer coated PLGA nanoparticles. This was a stark contrast to the 8% left in the blood stream after only 5 min when the dye was injected singularly. Others reported the ability of using polymeric micelles from chitosan and palmitic acid to encapsulate and protect an anti-cancer drug (i.e., tamoxifen) [148]. Tamoxifen release profiles showed a much more linear release when encapsulated in chitosan/palmitic acid micelles than with the free drug. Furthermore, polymeric micelles synthesized from phenylboronic acid-functionalized polycarbonate/PEG (PEG-PBC), urea-functionalized polycarbonate/PEG (PEG-PUC), and their diblock copolymers have been reported as drug delivery vehicles for an anti-fungal drug (i.e., amphotericin B) [149]. Result suggested that the PEG-PBC and diblock copolymers of PEG-PBC/PEG-PUC sustained the release of amphotericin B while PEG-PUC showed a burst release profile (Figure 5). Therefore, the use of polymeric micelles has demonstrated the ability to be a potential biomaterial for drug delivery and wound healing processes.

**Figure 6:**
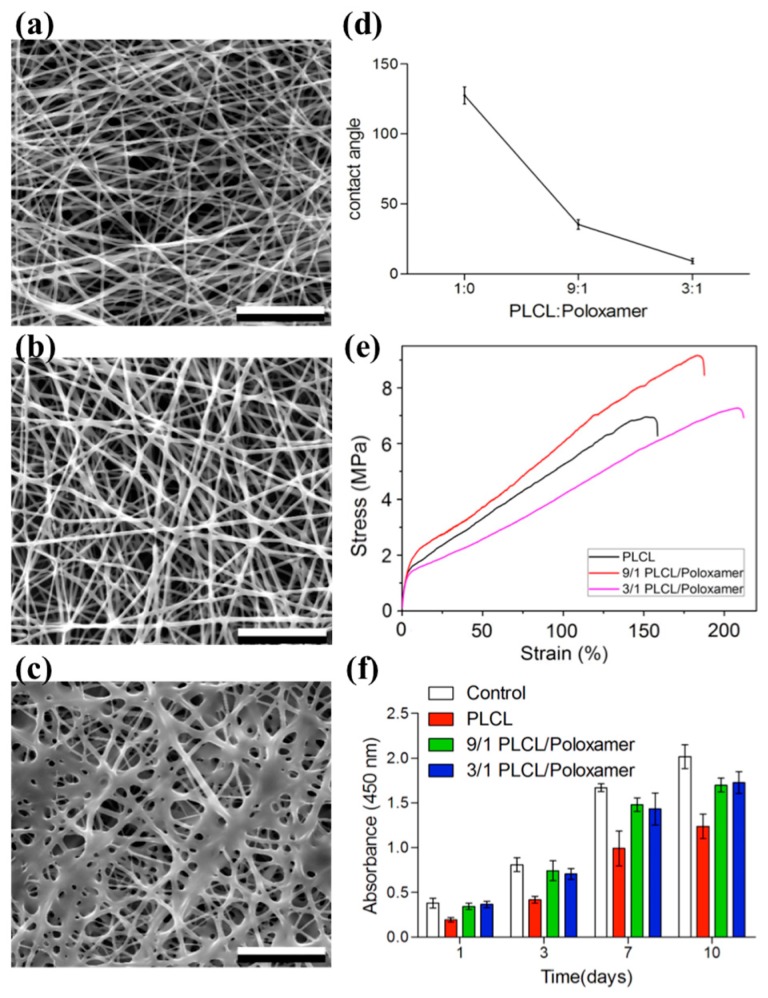
(**a**) SEM image of fiber structure from PLCL; (**b**) SEM image of fiber structure from PLCL/poloxamer (9/1 *w*/*w*); (**c**) SEM image of fiber structure from PLCL/poloxamer (3/1 *w*/*w*); (**d**) Water contact angle of PLCL/poloxamer fibers; (**e**) Stress strain curves of PLCL/poloxamer fibers; (**f**) Adipose-derived stem cell proliferation on PLCL/poloxamer fibers [150]. Figures were obtained from an open access article distributed under the Creative Commons Attribution License.

Studies have been conducted to explore the ability to incorporate polymeric micelles and/or nanoparticles in electrospun fibers for purposes of drug release and wound healing. Pan et al. demonstrated the biocompatibility of a bilayer scaffold electrospun from PLCL/poloxamer fibers and dextran/gelatin hydrogel (Figure 6) [150]. They found that the fiber scaffolds maintained cell viability and supported cell proliferation of adipose derived stem cells. Similarly, polymeric micelles have also been combined with other delivery systems such as hydrogels. Gong et al. compared the effects of drug release and wound healing characteristics on curcumin loaded PEG-PCL micelles with a combined micelle/hydrogel dressing [151]. The curcumin loaded PEG-PCL micelles showed a sustained drug release over 14 days and achieved a higher cumulative release than the micelle/hydrogel combination. In the in vivo model however, rats treated with the micelle/hydrogel combination showed higher tensile strength with a thicker epidermis during wound breaking testing. There was also an enhancement in wound closure rate using the micelle/hydrogel combination. These findings show the importance of selecting delivery vessels for drug-loaded micelles as the carriers can affect drug release rate and wound healing performance (Table 5). The use of polymeric micelles in drug delivery for wound healing is promising as they provide biocompatibility, extended drug release properties, and shorter healing time that make them ideal for future research.

### 7.2. Fiber-Nanoparticle Composites

Similarly, nanoparticles containing electrospun fibers have shown promising potential to make a significant impact in drug release and wound healing research. Studies demonstrated that gold, copper, titanium, and zinc have therapeutic effects during wound healing [153]. In addition, others have incorporated silver nanoparticles into electrospun fibers as an antibacterial agent [154,155]. For example, silver nanoparticles when incorporated with the bipolymer guar gum alkylamine exhibited faster wound healing rates and improved cosmetic attributes [156], whereas gold nanoparticles showed reduction in inflammatory response during the wound healing process [157]. In particular, Leu et al. showed that gold nanoparticles increased cell proliferation resulting in the reduction of wound healing time in mice [158]. Zinc nanoparticles exhibited antibacterial and positive effects on wound healing processes. Raguvaran et al. loaded zinc oxide nanoparticles into sodium alginate/gum acacia hydrogels and showed that zinc oxide at high levels can become toxic but at low levels had antibacterial and healing effects on wounds [159]. Martinez et al. demonstrated the beneficial effects of nitric oxide nanoparticles in wound healing, including antibacterial efficacy that promoted the regeneration of dermal architecture through protection of collagen from bacteria [160]. These examples suggest that nanoparticle provide therapeutic effect in wound healing.

Metallic nanoparticles have been combined with electrospun fibers for the purpose of wound healing. Rather et al. fabricated cerium oxide nanoparticles loaded PCL/Gelatin fibers to investigate the effects in wound healing [161]. The study focused on reducing levels of reactive oxygen species that may hinder proper wound healing when at an elevated level. In a similarly study, adhesive nanocomposite through immobilizing ultrasmall ceria nanocrystals onto the surface of uniform mesoporous silica nanoparticles showed the proper controlling of the reactive oxygen species and the ability to stimulate proliferation and cell migration using an in vivo mouse model [162]. The nanoparticle composite not only decreased healing time, but also reduced scar formation as well [163]. Polymeric nanoparticles have also been chosen for drug delivery for wound healing (Table 6). For example, chitosan based polymeric nanoparticles were fabricated in combination with PLLA-CL electrospun fibers to provide a dual delivery system for Nel-like molecule-1 growth factor [164]. Results indicated that the dual release system prolonged the release of growth factor when compared to plain PLLA-CL fibers. In vitro cell proliferation studies showed that human bone mesenchymal stem cells proliferated better on the dual delivery system than the fibers alone. In another study, chitosan, PVA, and zinc oxide composite nanoparticles displayed a much shorter healing time using an in vivo mouse model, whereas the composite nanoparticles showed almost no bacterial growth in antibacterial activity assay assessed by culturing pus from the wounds after three days of treatment [165]. Lipid nanocarriers have also been used to deliver drugs to wound sites. Sanad et al. used lipid nanocarriers in conjunction with a blended hylaruonic acid/chitosan fiber scaffold for the delivery of the natural diterpene lactone Andrographolide [166]. A prolonged release of Andrographolide, which has anti-inflammatory and antioxidant properties, was observed due to the effects of lipid nanocarrier coupled with the depolymerization of chitosan resulting in the reduction of wound healing time significantly.

## 8. Conclusions and Future Directions

Non-healing wounds remain a challenge for the development of dressing materials. Advances in nanotechnology enable the production of electrospun fibers, which have the potential to become the ideal candidate for encapsulation and delivery of small molecule drugs and/or large macromolecules to the wound site. In particular, electrospinning accepts most of the polymer and drugs where the interactions between them play in important role in drug release rates. For example, delivery of the coagulation factors and anti-inflammation drugs may be required for early stages of wound healing. The choice of using water-soluble (dissolution mechanism) polymers as well as those with minimal drug–polymer interactions (diffusion mechanism) facilitates the fast release of the drugs. In contrast, the proliferation and remodeling processes in late wound healing stages require the sustained delivery of the growth factors and genes. The use of blend fibers to enhance drug–polymer interactions, coaxial fibers to encapsulate drug in the core, and fiber composites will benefit the prolonged delivery of the biological agents. Therefore, the choice of using particular polymers and architectures in electrospun fibers will depend on the types of drugs and the stage of wound so that the healing process can be improved.

In this review, we explore the incorporation of various wound healing drugs, including small molecules, macromolecules, and gene vectors, in electrospun fibers and their release behaviors for wound healing. In addition, our review suggests that electrospun fibers are capable of integrating with typical small molecules, growth factors, and gene vectors to provide a sustained release behavior, depending on the polymer used. Furthermore, the incorporation of micelles/nanoparticles in fibers allows the formation of a composite material for multifunctional delivery purpose. While electrospinning possesses many advantages in drug delivery and tissue engineering, which are beneficial for wound healing, concerns over the use of harsh chemicals (cytotoxicity) may limit its use in pharmaceutical applications for dressing materials. In such case, exhaustion of the organic solvents under vacuum is required to eliminate residual chemicals that remain in the fibers after electrospinning. This is a costly and time-consuming step. Furthermore, low production rate (e.g., approximately 1~1.5 g/h via uniaxial electrospinning) can be another issue that limits the use of electrospun fibers in clinical aspect. This limitation has been improved by free-surface electrospinning process [167,168], whereas the production rates may be 5–10 fold higher than typical uniaxial electrospinning. In general, electrospun fibers demonstrate the ability of sustained release of small molecule drugs, macromolecules, and genes. This drug delivery platform is especially ideal for the use of topical dressing materials in wound healing applications.

## Figures and Tables

**Table 1:** Wound classifications and methods [4].

Classification Method	Subcategory	Characteristics	Examples
Time frame of healing	Acute	Faster healing (5–10 days)	Traumatic wounds, surgical wounds
	Chronic	Takes long time to heal	Leg ulceration
Wound closing method	Primary intention	Treated by closing the surface around the wound	Traumatic lacerations or surgical
	Secondary intentions	Treated by filling the gaps with granulating tissue	Leg ulcers, pressure damage, and lacerations
	Tertiary intention	Open intentionally to allow for drainage to take place	Abdominal wound
Wound tissue types	Black coloration	Shows black discoloration	Necrotic tissue
	Green	Shows green discoloration	Infected tissue
	Yellow	Shows yellow discoloration	Sloughy tissue
	Red	Shows red discoloration	Granulating tissue
	Pink	Shows pink discoloration	Epithelial tissue
Depth of wound	Superficial	Affect the epidermis	Abrasions
	Partial thickness	Affect both the epidermis and the inner dermal layer	Pressure sores and severe scale exits

**Table 2:** Representative natural and synthetic polymers used in electrospun fibers for wound healing and their corresponding electrospinning parameters.

Polymer(s) ^†^	Solvent(s)	Voltage (kV)	Distance (cm)	Flow Rate (mL/h)	Ref.
Natural					
Chitosan/PEO	50% Acetic Acid	15–35	15	0.1–2	[35]
Alginate/Soy Protein/PEO	Deionized Water	15	15	0.5	[49]
Gelatin	20% Acetic Acid	28–35	10	0.1–1	[50]
Cellulose	Acetic Acid	30–40	15	1	[39]
Collagen	PBS/Ethanol	18	15	0.3	[40]
Hyaluronic Acid/PCL	Formic Acid/Acetic Acid (75/25)	13	13	1	[51]
Keratin/PEO	88% Formic Acid	14	15	0.5	[52]
Silk Fibroin	Lithium Bromide	15	18	-	[53]
Synthetic					
PCL	Acedic Acid	9.5–22	15	0.15–1.2	[54]
PLGA/GT	1,1,1,3,3,3 hexafluoro-2-propanol	15	15	1	[55]
PU	*N*,*N*-dimethylformamide	35–45	10–15	0.5–1.5	[56]
PVDF	Dimethylformamide and Acetone	25	15	0.75	[57]
PVA/Silk Sericin	Deionized Water	8–12	20	3	[58]
PEO	Ethanol, Chloroform, and Deionized Water	13	10	3	[59]
PVP	Ethanol	15	10	1	[60]

^†^ PEO: Poly(ethylene oxide); PCL: Poly(*ε*-caprolactone); PLGA: Poly(lactic-*co*-glycolic acid); GT: Gum tragacanth; PU: Polyurethane; PVA: Polyvinyl alcohol; and PVP: Polyvinylpyrrolidone.

**Table 3:** Characteristics of small molecule drugs used in wound healing and their release behaviors from electrospun fibers.

Small Molecules Drugs	Agent		Fiber		Release (Units)			Ref.
	Aq. Sol. ^†^ (mg/mL)	Log P ^†^	Polymer(s) ^‡^	Loading (% *w*/*w*)	1 h	2 h	Others	
Hydrophilic								
Ciprofloxacin	1.35	−0.57	PVP	0.4	-	-	60% (1 min)	[81]
			PLCL/PDEGMA	10	12%	20%	80% (220 h)	[82]
			PVA/Alginate	-	30%	40%	85% (6 h)	[91]
Ampicillin	0.605	0.88	AL-BSA	5 10 20	23% 17% 7%	37% 25% 10%	99% (96 h) 81% (96 h) 40% (96 h)	[83]
			PMMA/Nylon6	1–20	-	-	30% (6 h) 50% (12 days)	[84]
			PCL	16.7	75%	80%	98% (24 h)	[85]
Captopril	4.52	1.02	PLLA PLGA PLCL	10 10 10	- - -	- - -	98%(48 h) 100% (48 h) 78% (48 h)	[92]
Metronidazole	5.92	-0.15	PCL	1–40	-	-	45% (1 day) 85% (5 days)	[86]
			PCL	4.8–14.4	20%	40%	90% (24 h)	[87]
			Chitosan/PEO	1 5 15	52% 70% 70%	75% 80% 100%	- - -	[88]
Cefazolin	0.487	−0.4	Chitosan/PEO	1	-	26%	65% (24 h)	[89]
			Gelatin	10	10%	30	95% (17 h)	[90]
Hydrophobic								
Asiaticoside	[93]	[93]	Alginate/PVA/Chitosan	2.5	20%	23%	83%(12 h)	[94]
Curcumin	0.006	3.62	PHBV	1 3 4.7	20% 55% 65%	40% 65% 67%	45% (5 h) 70% (5 h) 78% (5 h)	[95]
			PCL/GT	3	-	-	65% (20 days)	[96]
Ketoprofen	0.0213	3.29	PCL/Gelatin	5	-	-	40% (20 h) 80% (45 h)	[97]
			PVA	5	50%	-	62% (48 h)	[98]
			PNVCL-*co*-MAA	20	5%	-	35% (24 h)	[99]
			Cellulose Acetate	15	10%	-	60% (48 h)	[100]
Nifedipine	0.0177	2.49	Eudragit^®^	10	40%	50%	70% (8 h)	[101]
			PU	4.2	15%	-	75% (72 h)	[102]
			PNIPAAm/PU	12	8%	10%	23% (30 h)	[103]
Phenytoin	0.0711	2.26	PVA PCL PVA/PCL	2 2 2	27% 5% 11%	29% 8% 15%	88% (48 h) 16% (48 h) 47% (48 h)	[104]
Vancomycin	0.255	1.11	Alginate	10	10%	-	60% (48 h)	[105]
Methylene Blue	0.0296	3.61	PHB/PEG	-	32%	-	90% (7 days)	[106]

^†^ *DrugBank v5.0.10*: Calculated using ALOGPS v2.1; (accessed 30 November 2017); ^‡^ PVP: Polyvinylpyrrolidone; PLCL: Poly(lactic-*co*-ε-caprolactone); PDEGMA: Poly(di(ethylene glycol) methyl ether methacrylate); PVA: Polyvinyl alcohol; AL-BSA: Amyloid-like bovine serum albumin; PMMA: Poly(methyl methacrylate); PCL: Poly(*ε*-caprolactone); PLLA: Poly(l-lactic acid); PLGA: Poly(lactic-*co*-glycolic acid); PEO: Poly(ethylene oxide); PHBV: Poly(3-hydroxybutyric acid-co-3-hydroxyvaleric acid); GT: Gum tragacanth; PNVCL-*co*-MAA: Poly(*N*-vinylcaprolactam-*co*-methacrylic acid); PU: Polyurethane; PNIPAAm: Poly(*N*-isopropylacrylamide); PHB: Poly(R-3-hydroxybutyrate); and PEG: Polyethylene glycol.

**Table 4:** The use of growth factors in electrospun fibers for wound healing applications with respect to solvent used during electrospinning, types of cells studies, and methods to incorporate growth factors in/on fibers.

Growth Factor	Polymer	Solvent	Cell	Method	Ref.
EGF	PCL and PCL–PEG/PCL	Methanol/Chloroform	Human Primary Keratinocyte	Immobilization	[119]
	PLGA and Gelatin	Chloroform/Acetone and Acetic Acid	Human Fibroblasts	Emulsion	[120]
	Silk Fibroin	Lithium Bromide	Human Dermal Fibroblasts	Blend	[121]
	PCL and PCL/Collagen	DMF/DCM and HFIP	Human Dermal Keratinocyte	Immobilization	[122]
	Gelatin/PLA-*co*-PCL	HFIP	Human Dermal Fibroblasts	Coaxial	[123] [124]
	Silk/PEO	Lithium Bromide	-	Blend/Coating	[125]
bFGF/EGF	PCL-PEG	Methanol and Chloroform	Keratinocyte and fibroblast	Coaxial/Immobilization	[112]
	PLGA/PEO	Chloroform and DMF/Water	Human Skin Fibroblasts	Fiber containing GFs encapsulated microspheres.	[126]
bFGF, EGF, VEGF, PDGF	Collagen-Hyaluronic Acid/Gelatin Nanoparticle	Hyaluronic Acid: NaOH/DMF Collagen: Acetic Acid	HUVEC	Blend: bEGF/EGF In nanoparticle: VEGF/PDGF	[111]
PDGF	PCL/Collagen/Hyaluronic Acid	HFP, PBS	MSC	Blend	[113]
FGF2	PHBV, PEO	2, 2, 2-trifluoroethanol	MSC	FGF2-miR-218 induction on aligned PHBV fibers	[115]
KGF	PLA/PCL	Chloroform, Acetone	Fibroblasts	Seeded scaffolds with mouse fibroblast in DMEM with FBS	[127]
TGF-β	MeHA, HH, PCL, HA	DI Water	Cartilage	Composite scaffolds of HA and PCL with TGFβ3	[114]
VEGF	PLGA	Water-in-oil emulsions, Dichloromethane, PBS, BSA	HUVEC, Endothelials	PVEES, and NVEES Scaffolds containing VEGF	[128]
GM-CSF	Chitosan	HCl	In vivo mouse model	Hydrogels containing ovalbumin and GM-CSF	[117]
CTGF	PCL	Chloroform	MSC	Aligned fibers as a guide	[118]

**Table 5:** Summary of polymeric micelles used in wound healing.

Polymeric Micelles	Drug	Functions	Ref.
Poloxamer 407 and 908/PLGA nanoparticles	Rose Bengal Dye	Showed protective effects of Poloxamer 407 and 908 micelles.	[147]
Chitosan/Palmitic Acid	Tamoxifen	Release profiles showed much more linear release when encapsulated in micelle structures.	[148]
phenylboronic acid-functionalized polycarbonate/PEG (PEG-PBC)/urea-functionalized polycarbonate/PEG (PEG-PUC)/diblock copolymers	Amphotericin B	Used to study delivery of anti-fungal medication. PEG-PBC and diblock copolymers of PEG-PBC and PEG-PUC showed sustained release of drug while PEG-PUC had burst release profile.	[149]
Poly(l-aspartic acid)-*b*-poly(ethylene glycol)-*b*-poly(l-aspartic acid) (PLD-PEG-PLD)	Doxorubicin	Showed effect pH of release media has on release profiles of doxorubicin loaded PLD-PEG-PLD micelles. Found more acidic environment correlated to higher release rates.	[152]
PLCL/poloxamer with dextran/gelatin hydrogel	No Drug	Showed fibers supported cell viability and proliferation when tested with stem cells. Mechanical properties increased with addition of of Poloxamer at 9/1 ratio.	[150]
PEG-PCL and PEG-PCL/hydrogel	Curcumin	Micelle structure sustained release 14 days and achieved higher cumulative release rate than micelle/hydrogel. In Vivo model showed micelle. Hydrogel combination produced higher tensile strength and thicker epidermis during wound healing breaking test. Micelle/Hydrogel also showed enhanced wound closure rate.	[151]

**Table 6:** Summary of nanoparticles used in wound healing on their effects and functions.

Nanoparticles	Effects	Functions	Ref.
Silver/guar gum alkylamine	Antibacterial	Exhibited faster would healing rates and improved cosmetic attributes.	[156]
Gold	Anti-Inflammatory	Wounds exhibited reduction in inflammatory response. Increase in cell proliferation resulting in reduction of wound healing time in mice.	[157]
Zinc Oxide loaded alginate/gun acacia	Antibacterial	Showed that Zinc nanoparticles have antibacterial effects at low levels but can become toxic at high levels.	[159]
Nitric Oxide	Antibacterial	Promoted regeneration of dermal architecture through protection of collagen from bacteria.	[160]
Cerum Oxide loaded PCL/Gelatin fibers	Reduction of reactive oxygen levels, decreased healing time	Lowered the level of reactive oxygen levels that hinder proper wound healing.	[161]
Adhesive nanocomposite made of ultrasmall ceria nanocrystals adhered to the surface of mesoporous silica nanoparticles	Reduction of reactive oxygen levels, decreased healing time	Reduced healing time and scar formation. Stimulated proliferation and cell migration in vivo.	[163]
Chitosan nanoparticles with PLLA-CL fibers	Nel-like mlecule-1 growth factor delivery	Dual release system prolonged release of growth factor when compared to plain PLLA-CL fibers. Dual release system Increased cell proliferation in human bone mesenchymal stem cells.	[164]
Chitosan/PVA/Zinc Oxide	Decreased wound healing time/Antibacterial	Displayed shorter healing time and almost no bacterial growth in cultured pus from wounds.	[165]
Lipid nanocarrier/Hyaluronic Acid/Chitosan	Drug delivery	Prolonged release of Andrographolide combined with depolymerization of chitosan resulted in the reduction of wound healing time.	[166]

## References

[1] Sidgwick G.P., McGeorge D., Bayat A. (2015). A comprehensive evidence-based review on the role of topicals and dressings in the management of skin scarring. Arch. Dermatol. Res..

[2] Chou S.-F., Carson D., Woodrow K.A. (2015). Current strategies for sustaining drug release from electrospun nanofibers. J. Control. Release.

[3] Strodtbeck F. (2001). Physiology of wound healing. Newborn Infant Nurs. Rev..

[4] Merlin-Manton E. (2017). Wound care: Selecting the right dressings. Pract. Nurse.

[5] Tejiram S., Kavalukas S.L., Shupp J.W., Barbul A., Ågren M.S. (2016). 1-Wound healing. Wound Healing Biomaterials.

[6] Velnar T., Bailey T., Smrkolj V. (2009). The wound healing process: An overview of the cellular and molecular mechanisms. J. Int. Med. Res..

[7] Abrigo M., McArthur S.L., Kingshott P. (2014). Electrospun nanofibers as dressings for chronic wound care: Advances, challenges, and future prospects. Macromol. Biosci..

[8] Whitney J.D. (2005). Overview: Acute and Chronic Wounds. Nurs. Clin. N. Am..

[9] Boateng J.S., Matthews K.H., Stevens H.N.E., Eccleston G.M. (2008). Wound healing dressings and drug delivery systems: A review. J. Pharm. Sci..

[10] Arasteh S., Kazemnejad S., Khanjani S., Heidari-Vala H., Akhondi M.M., Mobini S. (2016). Fabrication and characterization of nano-fibrous bilayer composite for skin regeneration application. Methods.

[11] Lee Y.-H., Chang J.-J., Yang M.-C., Chien C.-T., Lai W.-F. (2012). Acceleration of wound healing in diabetic rats by layered hydrogel dressing. Carbohydr. Polym..

[12] Kasuya A., Tokura Y. (2014). Attempts to accelerate wound healing. J. Dermatol. Sci..

[13] Neuenschwander P.F., Jesty J. (2011). Blood coagulation. Encyclopedia of Life Sciences.

[14] Castellanos G., Bernabé-García Á., Moraleda J.M., Nicolás F.J. (2017). Amniotic membrane application for the healing of chronic wounds and ulcers. Placenta.

[15] Hosgood G. (2006). Stages of Wound healing and their clinical relevance. Vet. Clin. N. Am. Small Anim. Pract..

[16] Reinke J.M., Sorg H. (2012). Wound repair and regeneration. Eur. Surg. Res..

[17] Landén N.X., Li D., Ståhle M. (2016). Transition from inflammation to proliferation: A critical step during wound healing. Cell. Mol. Life Sci..

[18] Clinical Guidelines (Nursing): Wound Care. https://www.rch.org.au/rchcpg/hospital_clinical_guideline_index/Wound_care/.

[19] Hanna J.R., Giacopelli J.A. (1997). A review of wound healing and wound dressing products. J. Foot Ankle Surg..

[20] Harding K.G., Morris H.L., Patel G.K. (2002). Healing chronic wounds. BMJ Br. Med. J. Lond..

[21] Dovi J.V., Szpaderska A.M., DiPietro L.A. (2004). Neutrophil function in the healing wound: Adding insult to injury?. Thromb. Haemost..

[22] Mathews V., Hanson P.T., Ford E., Fujita J., Polonsky K.S., Graubert T.A. (2004). Recruitment of bone marrow-derived endothelial cells to sites of pancreatic β-cell injury. Diabetes.

[23] Velazquez O.C. (2007). Angiogenesis and vasculogenesis: Inducing the growth of new blood vessels and wound healing by stimulation of bone marrow–derived progenitor cell mobilization and homing. J. Vasc. Surg..

[24] Aramwit P., Ågren M.S. (2016). 1-Introduction to biomaterials for wound healing. Wound Healing Biomaterials.

[25] Sood A., Granick M.S., Tomaselli N.L. (2014). Wound dressings and comparative effectiveness data. Adv. Wound Care.

[26] Liu M., Duan X.-P., Li Y.-M., Yang D.-P., Long Y.-Z. (2017). Electrospun nanofibers for wound healing. Mater. Sci. Eng. C.

[27] Wang J., Windbergs M. (2017). Functional electrospun fibers for the treatment of human skin wounds. Eur. J. Pharm. Biopharm..

[28] Ahmadi Majd S., Rabbani Khorasgani M., Moshtaghian S.J., Talebi A., Khezri M. (2016). Application of Chitosan/PVA Nano fiber as a potential wound dressing for streptozotocin-induced diabetic rats. Int. J. Biol. Macromol..

[29] Chou S.-F., Woodrow K.A. (2017). Relationships between mechanical properties and drug release from electrospun fibers of PCL and PLGA blends. J. Mech. Behav. Biomed. Mater..

[30] Chen S., Boda S.K., Batra S.K., Li X., Xie J. (2017). Emerging roles of electrospun nanofibers in cancer research. Adv. Healthc. Mater..

[31] Mogoşanu G.D., Grumezescu A.M. (2014). Natural and synthetic polymers for wounds and burns dressing. Int. J. Pharm..

[32] Huang S., Fu X. (2010). Naturally derived materials-based cell and drug delivery systems in skin regeneration. J. Control. Release.

[33] Dai T., Tanaka M., Huang Y.-Y., Hamblin M.R. (2011). Chitosan preparations for wounds and burns: Antimicrobial and wound-healing effects. Expert Rev. Anti-Infect. Ther..

[34] Bano I., Arshad M., Yasin T., Ghauri M.A., Younus M. (2017). Chitosan: A potential biopolymer for wound management. Int. J. Biol. Macromol..

[35] Pakravan M., Heuzey M.-C., Ajji A. (2011). A fundamental study of chitosan/PEO electrospinning. Polymer.

[36] Lu J.-W., Zhu Y.-L., Guo Z.-X., Hu P., Yu J. (2006). Electrospinning of sodium alginate with poly(ethylene oxide). Polymer.

[37] Topuz F., Uyar T. (2017). Electrospinning of gelatin with tunable fiber morphology from round to flat/ribbon. Mater. Sci. Eng. C.

[38] Ostrovidov S., Shi X., Zhang L., Liang X., Kim S.B., Fujie T., Ramalingam M., Chen M., Nakajima K., Al-Hazmi F. (2014). Myotube formation on gelatin nanofibers—Multi-walled carbon nanotubes hybrid scaffolds. Biomaterials.

[39] Zhang K., Li Z., Kang W., Deng N., Yan J., Ju J., Liu Y., Cheng B. (2018). Preparation and characterization of tree-like cellulose nanofiber membranes via the electrospinning method. Carbohydr. Polym..

[40] Bak S.Y., Yoon G.J., Lee S.W., Kim H.W. (2016). Effect of humidity and benign solvent composition on electrospinning of collagen nanofibrous sheets. Mater. Lett..

[41] Sadeghi-Avalshahr A., Nokhasteh S., Molavi A.M., Khorsand-Ghayeni M., Mahdavi-Shahri M. (2017). Synthesis and characterization of collagen/PLGA biodegradable skin scaffold fibers. Regen. Biomater..

[42] Kutlusoy T., Oktay B., Apohan N.K., Süleymanoğlu M., Kuruca S.E. (2017). Chitosan-co-Hyaluronic acid porous cryogels and their application in tissue engineering. Int. J. Biol. Macromol..

[43] Liu Y., Ma G., Fang D., Xu J., Zhang H., Nie J. (2011). Effects of solution properties and electric field on the electrospinning of hyaluronic acid. Carbohydr. Polym..

[44] Brenner E.K., Schiffman J.D., Thompson E.A., Toth L.J., Schauer C.L. (2012). Electrospinning of hyaluronic acid nanofibers from aqueous ammonium solutions. Carbohydr. Polym..

[45] Esparza Y., Ullah A., Boluk Y., Wu J. (2017). Preparation and characterization of thermally crosslinked poly(vinyl alcohol)/feather keratin nanofiber scaffolds. Mater. Des..

[46] Yukseloglu S.M., Sokmen N., Canoglu S. (2015). Biomaterial applications of silk fibroin electrospun nanofibres. Microelectron. Eng..

[47] Dias Antunes M., da Silva Dannenberg G., Fiorentini Â.M., Pinto V.Z., Lim L.-T., da Rosa Zavareze E., Dias A.R.G. (2017). Antimicrobial electrospun ultrafine fibers from zein containing eucalyptus essential oil/cyclodextrin inclusion complex. Int. J. Biol. Macromol..

[48] Deng L., Kang X., Liu Y., Feng F., Zhang H. (2018). Characterization of gelatin/zein films fabricated by electrospinning vs solvent casting. Food Hydrocoll..

[49] Wongkanya R., Chuysinuan P., Pengsuk C., Techasakul S., Lirdprapamongkol K., Svasti J., Nooeaid P. (2017). Electrospinning of alginate/soy protein isolated nanofibers and their release characteristics for biomedical applications. J. Sci. Adv. Mater. Devices.

[50] Okutan N., Terzi P., Altay F. (2014). Affecting parameters on electrospinning process and characterization of electrospun gelatin nanofibers. Food Hydrocoll..

[51] Entekhabi E., Haghbin Nazarpak M., Moztarzadeh F., Sadeghi A. (2016). Design and manufacture of neural tissue engineering scaffolds using hyaluronic acid and polycaprolactone nanofibers with controlled porosity. Mater. Sci. Eng. C.

[52] Ma H., Shen J., Cao J., Wang D., Yue B., Mao Z., Wu W., Zhang H. (2017). Fabrication of wool keratin/polyethylene oxide nano-membrane from wool fabric waste. J. Clean. Prod..

[53] Kishimoto Y., Morikawa H., Yamanaka S., Tamada Y. (2017). Electrospinning of silk fibroin from all aqueous solution at low concentration. Mater. Sci. Eng. C.

[54] Tampau A., González-Martinez C., Chiralt A. (2017). Carvacrol encapsulation in starch or PCL based matrices by electrospinning. J. Food Eng..

[55] Ranjbar-Mohammadi M., Zamani M., Prabhakaran M.P., Bahrami S.H., Ramakrishna S. (2016). Electrospinning of PLGA/gum tragacanth nanofibers containing tetracycline hydrochloride for periodontal regeneration. Mater. Sci. Eng. C.

[56] Ju J., Shi Z., Fan L., Liang Y., Kang W., Cheng B. (2017). Preparation of elastomeric tree-like nanofiber membranes using thermoplastic polyurethane by one-step electrospinning. Mater. Lett..

[57] Dorneanu P.P., Cojocaru C., Olaru N., Samoila P., Airinei A., Sacarescu L. (2017). Electrospun PVDF fibers and a novel PVDF/CoFe_2_O_4_ fibrous composite as nanostructured sorbent materials for oil spill cleanup. Appl. Surf. Sci..

[58] Yan S., Li X., Dai J., Wang Y., Wang B., Lu Y., Shi J., Huang P., Gong J., Yao Y. (2017). Electrospinning of PVA/sericin nanofiber and the effect on epithelial-mesenchymal transition of A549 cells. Mater. Sci. Eng. C.

[59] Son W.K., Youk J.H., Lee T.S., Park W.H. (2004). The effects of solution properties and polyelectrolyte on electrospinning of ultrafine poly(ethylene oxide) fibers. Polymer.

[60] Reksamunandar R.P., Edikresnha D., Munir M.M., Damayanti S. (2017). Khairurrijal Encapsulation of β-carotene in poly(vinylpyrrolidone) (PVP) by Electrospinning Technique. Procedia Eng..

[61] Chou S.-F., Gunaseelan S., Kiellani M.H.H., Thottempudi V.V.K., Neuenschwander P., Nie H. (2017). A review of injectable and implantable biomaterials for treatment and repair of soft tissues in wound healing. J. Nanotechnol..

[62] Ponjavic M., Nikolic M.S., Nikodinovic-Runic J., Jeremic S., Stevanovic S., Djonlagic J. (2017). Degradation behaviour of PCL/PEO/PCL and PCL/PEO block copolymers under controlled hydrolytic, enzymatic and composting conditions. Polym. Test..

[63] Luong-Van E., Grøndahl L., Chua K.N., Leong K.W., Nurcombe V., Cool S.M. (2006). Controlled release of heparin from poly(ε-caprolactone) electrospun fibers. Biomaterials.

[64] Lu L., Garcia C.A., Mikos A.G. (1999). In vitro degradation of thin poly(DL-lactic-*co*-glycolic acid) films. J. Biomed. Mater. Res..

[65] Park T.G. (1995). Degradation of poly(lactic-co-glycolic acid) microspheres: Effect of copolymer composition. Biomaterials.

[66] Peña J., Corrales T., Izquierdo-Barba I., Doadrio A.L., Vallet-Regí M. (2006). Long term degradation of poly(ε-caprolactone) films in biologically related fluids. Polym. Degrad. Stab..

[67] You Y., Min B.-M., Lee S.J., Lee T.S., Park W.H. (2005). In vitro degradation behavior of electrospun polyglycolide, polylactide, and poly(lactide-co-glycolide). J. Appl. Polym. Sci..

[68] Yeganegi M., Kandel R.A., Santerre J.P. (2010). Characterization of a biodegradable electrospun polyurethane nanofiber scaffold: Mechanical properties and cytotoxicity. Acta Biomater..

[69] Sheikh F.A., Zargar M.A., Tamboli A.H., Kim H. (2016). A super hydrophilic modification of poly(vinylidene fluoride) (PVDF) nanofibers: By in situ hydrothermal approach. Appl. Surf. Sci..

[70] Li X., Kanjwal M.A., Lin L., Chronakis I.S. (2013). Electrospun polyvinyl-alcohol nanofibers as oral fast-dissolving delivery system of caffeine and riboflavin. Colloids Surf. B Biointerfaces.

[71] Kim T.G., Lee D.S., Park T.G. (2007). Controlled protein release from electrospun biodegradable fiber mesh composed of poly(ε-caprolactone) and poly(ethylene oxide). Int. J. Pharm..

[72] Bui H.T., Chung O.H., Cruz J.D., Park J.S. (2014). Fabrication and characterization of electrospun curcumin-loaded polycaprolactone-polyethylene glycol nanofibers for enhanced wound healing. Macromol. Res..

[73] Ahire J.J., Robertson D.D., van Reenen A.J., Dicks L.M.T. (2017). Polyethylene oxide (PEO)-hyaluronic acid (HA) nanofibers with kanamycin inhibits the growth of Listeria monocytogenes. Biomed. Pharmacother..

[74] Balogh A., Farkas B., Verreck G., Mensch J., Borbás E., Nagy B., Marosi G., Nagy Z.K. (2016). AC and DC electrospinning of hydroxypropylmethylcellulose with polyethylene oxides as secondary polymer for improved drug dissolution. Int. J. Pharm..

[75] Haider A., Haider S., Kang I.-K. (2015). A comprehensive review summarizing the effect of electrospinning parameters and potential applications of nanofibers in biomedical and biotechnology. Arab. J. Chem..

[76] Megelski S., Stephens J.S., Chase D.B., Rabolt J.F. (2002). Micro- and nanostructured surface morphology on electrospun polymer fibers. Macromolecules.

[77] Rodoplu D., Mutlu M. (2012). Effects of electrospinning setup and process parameters on nanofiber morphology intended for the modification of quartz crystal microbalance surfaces. J. Eng. Fibers Fabr..

[78] Matabola K.P., Moutloali R.M. (2013). The influence of electrospinning parameters on the morphology and diameter of poly(vinyledene fluoride) nanofibers- effect of sodium chloride. J. Mater. Sci..

[79] Ball C., Chou S.-F., Jiang Y., Woodrow K.A. (2016). Coaxially electrospun fiber-based microbicides facilitate broadly tunable release of maraviroc. Mater. Sci. Eng. C.

[80] Zamani M., Prabhakaran M.P., Ramakrishna S. (2013). Advances in drug delivery via electrospun and electrosprayed nanomaterials. Int. J. Nanomed..

[81] Contardi M., Heredia-Guerrero J.A., Perotto G., Valentini P., Pompa P.P., Spanò R., Goldoni L., Bertorelli R., Athanassiou A., Bayer I.S. (2017). Transparent ciprofloxacin-povidone antibiotic films and nanofiber mats as potential skin and wound care dressings. Eur. J. Pharm. Sci..

[82] Li H., Williams G.R., Wu J., Lv Y., Sun X., Wu H., Zhu L.-M. (2017). Thermosensitive nanofibers loaded with ciprofloxacin as antibacterial wound dressing materials. Int. J. Pharm..

[83] Kabay G., Meydan A.E., Kaleli Can G., Demirci C., Mutlu M. (2017). Controlled release of a hydrophilic drug from electrospun amyloid-like protein blend nanofibers. Mater. Sci. Eng. C.

[84] Sohrabi A., Shaibani P.M., Etayash H., Kaur K., Thundat T. (2013). Sustained drug release and antibacterial activity of ampicillin incorporated poly(methyl methacrylate)–nylon6 core/shell nanofibers. Polymer.

[85] Sultanova Z., Kaleli G., Kabay G., Mutlu M. (2016). Controlled release of a hydrophilic drug from coaxially electrospun polycaprolactone nanofibers. Int. J. Pharm..

[86] Xue J., He M., Niu Y., Liu H., Crawford A., Coates P., Chen D., Shi R., Zhang L. (2014). Preparation and in vivo efficient anti-infection property of GTR/GBR implant made by metronidazole loaded electrospun polycaprolactone nanofiber membrane. Int. J. Pharm..

[87] He M., Jiang H., Wang R., Xie Y., Zhao C. (2017). Fabrication of metronidazole loaded poly (ε-caprolactone)/zein core/shell nanofiber membranes via coaxial electrospinning for guided tissue regeneration. J. Colloid Interface Sci..

[88] Zupančič Š., Potrč T., Baumgartner S., Kocbek P., Kristl J. (2016). Formulation and evaluation of chitosan/polyethylene oxide nanofibers loaded with metronidazole for local infections. Eur. J. Pharm. Sci..

[89] Sadri M., Sorkhi S.A. (2017). Preparation and characterization of CS/PEO/cefazolin nanofibers with in vitro and in vivo testing. Nanomedicine Res. J..

[90] Rath G., Hussain T., Chauhan G., Garg T., Goyal A.K. (2016). Development and characterization of cefazolin loaded zinc oxide nanoparticles composite gelatin nanofiber mats for postoperative surgical wounds. Mater. Sci. Eng. C.

[91] Kataria K., Gupta A., Rath G., Mathur R.B., Dhakate S.R. (2014). In vivo wound healing performance of drug loaded electrospun composite nanofibers transdermal patch. Int. J. Pharm..

[92] Zhang H., Lou S., Williams G.R., Branford-White C., Nie H., Quan J., Zhu L.-M. (2012). A systematic study of captopril-loaded polyester fiber mats prepared by electrospinning. Int. J. Pharm..

[93] Zheng X.-F., Lu X.-Y. (2011). Measurement and correlation of solubilities of asiaticoside in water, methanol, ethanol, *n* -propanol, *n* -butanol, and a methanol + water mixture from (278.15 to 343.15) K. J. Chem. Eng. Data.

[94] Zhu L., Liu X., Du L., Jin Y. (2016). Preparation of asiaticoside-loaded coaxially electrospinning nanofibers and their effect on deep partial-thickness burn injury. Biomed. Pharmacother..

[95] Mutlu G., Calamak S., Ulubayram K., Guven E. (2018). Curcumin-loaded electrospun PHBV nanofibers as potential wound-dressing material. J. Drug Deliv. Sci. Technol..

[96] Ranjbar-Mohammadi M., Rabbani S., Bahrami S.H., Joghataei M.T., Moayer F. (2016). Antibacterial performance and in vivo diabetic wound healing of curcumin loaded gum tragacanth/poly(ε-caprolactone) electrospun nanofibers. Mater. Sci. Eng. C.

[97] Basar A.O., Castro S., Torres-Giner S., Lagaron J.M., Turkoglu Sasmazel H. (2017). Novel poly(ε-caprolactone)/gelatin wound dressings prepared by emulsion electrospinning with controlled release capacity of Ketoprofen anti-inflammatory drug. Mater. Sci. Eng. C.

[98] Kenawy E.-R., Abdel-Hay F.I., El-Newehy M.H., Wnek G.E. (2007). Controlled release of ketoprofen from electrospun poly(vinyl alcohol) nanofibers. Mater. Sci. Eng. A.

[99] Liu L., Bai S., Yang H., Li S., Quan J., Zhu L., Nie H. (2016). Controlled release from thermo-sensitive PNVCL-*co*-MAA electrospun nanofibers: The effects of hydrophilicity/hydrophobicity of a drug. Mater. Sci. Eng. C.

[100] Yu D.-G., Yu J.-H., Chen L., Williams G.R., Wang X. (2012). Modified coaxial electrospinning for the preparation of high-quality ketoprofen-loaded cellulose acetate nanofibers. Carbohydr. Polym..

[101] Hamori M., Yoshimatsu S., Hukuchi Y., Shimizu Y., Fukushima K., Sugioka N., Nishimura A., Shibata N. (2014). Preparation and pharmaceutical evaluation of nano-fiber matrix supported drug delivery system using the solvent-based electrospinning method. Int. J. Pharm..

[102] Lin X., Tang D., Du H. (2013). Self-assembly and controlled release behaviour of the water-insoluble drug nifedipine from electrospun PCL-based polyurethane nanofibres: Self-assembly and release of drug. J. Pharm. Pharmacol..

[103] Lin X., Tang D., Gu S., Du H., Jiang E. (2013). Electrospun poly(*N*-isopropylacrylamide)/poly(caprolactone)-based polyurethane nanofibers as drug carriers and temperature-controlled release. New J. Chem..

[104] Zahedi P., Rezaeian I., Jafari S.H. (2013). In vitro and in vivo evaluations of phenytoin sodium-loaded electrospun PVA, PCL, and their hybrid nanofibrous mats for use as active wound dressings. J. Mater. Sci..

[105] Kurczewska J., Pecyna P., Ratajczak M., Gajęcka M., Schroeder G. (2017). Halloysite nanotubes as carriers of vancomycin in alginate-based wound dressing. Saudi Pharm. J..

[106] El-Khordagui L., El-Sayed N., Galal S., El-Gowelli H., Omar H., Mohamed M. (2017). Photosensitizer-eluting nanofibers for enhanced photodynamic therapy of wounds: A preclinical study in immunocompromized rats. Int. J. Pharm..

[107] Anstead G.M., Hart L.M., Sunahara J.F., Liter M.E. (1996). Phenytoin in wound healing. Ann. Pharmacother..

[108] Hokkam E., El-Labban G., Shams M., Rifaat S., El-mezaien M. (2011). The use of topical phenytoin for healing of chronic venous ulcerations. Int. J. Surg..

[109] Lupo E., Locher R., Weisser B., Vetter W. (1994). In vitro antioxidant activity of calcium antagonists against LDL oxidation compared with α-tocopherol. Biochem. Biophys. Res. Commun..

[110] Barrientos S., Stojadinovic O., Golinko M.S., Brem H., Tomic-Canic M. (2008). PERSPECTIVE ARTICLE: Growth factors and cytokines in wound healing. Wound Repair Regen..

[111] Lai H.-J., Kuan C.-H., Wu H.-C., Tsai J.-C., Chen T.-M., Hsieh D.-J., Wang T.-W. (2014). Tailored design of electrospun composite nanofibers with staged release of multiple angiogenic growth factors for chronic wound healing. Acta Biomater..

[112] Choi J.S., Choi S.H., Yoo H.S. (2011). Coaxial electrospun nanofibers for treatment of diabetic ulcers with binary release of multiple growth factors. J. Mater. Chem..

[113] Phipps M., Ma Y., Bellis S. (2012). Delivery of platelet-derived growth factor as a chemotactic factor for mesenchymal stem cells by bone-mimetic electrospun scaffolds. PLoS ONE.

[114] Kim I.L., Pfeifer C.G., Fisher M.B., Saxena V., Meloni G.R., Kwon M.Y., Kim M., Steinberg D.R., Mauck R.L., Burdick J.A. (2015). Fibrous scaffolds with varied fiber chemistry and growth factor delivery promote repair in a porcine cartilage defect model. Tissue Eng. Part A.

[115] Hu F., Zhang X., Liu H., Xu P., Doulathunnisa, Teng G., Xiao Z. (2017). Neuronally differentiated adipose-derived stem cells and aligned PHBV nanofiber nerve scaffolds promote sciatic nerve regeneration. Biochem. Biophys. Res. Commun..

[116] Davis M.E., Hsieh P.C.H., Takahashi T., Song Q., Zhang S., Kamm R.D., Grodzinsky A.J., Anversa P., Lee R.T. (2006). Local myocardial insulin-like growth factor 1 (IGF-1) delivery with biotinylated peptide nanofibers improves cell therapy for myocardial infarction. Proc. Natl. Acad. Sci. USA.

[117] Noh K.H., Park Y.M., Kim H.S., Kang T.H., Song K.-H., Lee Y.-H., Byeon Y., Jeon H.N., Jung I.D., Shin B.C. (2014). GM-CSF-loaded chitosan hydrogel as an immunoadjuvant enhances antigen-specific immune responses with reduced toxicity. BMC Immunol..

[118] Olvera D., Sathy B.N., Carroll S.F., Kelly D.J. (2017). Modulating microfibrillar alignment and growth factor stimulation to regulate mesenchymal stem cell differentiation. Acta Biomater..

[119] Choi J.S., Leong K.W., Yoo H.S. (2008). In vivo wound healing of diabetic ulcers using electrospun nanofibers immobilized with human epidermal growth factor (EGF). Biomaterials.

[120] Norouzi M., Shabani I., Ahvaz H.H., Soleimani M. (2015). PLGA/gelatin hybrid nanofibrous scaffolds encapsulating EGF for skin regeneration. J. Biomed. Mater. Res. A.

[121] Schneider A., Wang X.Y., Kaplan D.L., Garlick J.A., Egles C. (2009). Biofunctionalized electrospun silk mats as a topical bioactive dressing for accelerated wound healing. Acta Biomater..

[122] Gümüşderelioğlu M., Dalkıranoğlu S., Aydın R.S.T., Çakmak S. (2011). A novel dermal substitute based on biofunctionalized electrospun PCL nanofibrous matrix. J. Biomed. Mater. Res. A.

[123] Jin G., Prabhakaran M.P., Ramakrishna S. (2014). Photosensitive and biomimetic core–shell nanofibrous scaffolds as wound dressing. Photochem. Photobiol..

[124] Jin G., Prabhakaran M.P., Kai D., Ramakrishna S. (2013). Controlled release of multiple epidermal induction factors through core–shell nanofibers for skin regeneration. Eur. J. Pharm. Biopharm..

[125] Gil E.S., Panilaitis B., Bellas E., Kaplan D.L. (2013). Functionalized silk biomaterials for wound healing. Adv. Healthc. Mater..

[126] Mirdailami O., Soleimani M., Dinarvand R., Khoshayand M.R., Norouzi M., Hajarizadeh A., Dodel M., Atyabi F. (2015). Controlled release of rhEGF and rhbFGF from electrospun scaffolds for skin regeneration. J. Biomed. Mater. Res. A.

[127] Kobsa S., Kristofik N.J., Sawyer A.J., Bothwell A.L.M., Kyriakides T.R., Saltzman W.M. (2013). An electrospun scaffold integrating nucleic acid delivery for treatment of full thickness wounds. Biomaterials.

[128] Zhao Q., Lu W.W., Wang M. (2017). Modulating the release of vascular endothelial growth factor by negative-voltage emulsion electrospinning for improved vascular regeneration. Mater. Lett..

[129] Song D.W., Kim S.H., Kim H.H., Lee K.H., Ki C.S., Park Y.H. (2016). Multi-biofunction of antimicrobial peptide-immobilized silk fibroin nanofiber membrane: Implications for wound healing. Acta Biomater..

[130] Sebe I., Ostorhazi E., Fekete A., Kovacs K.N., Zelko R., Kovalszky I., Li W., Wade J.D., Szabo D., Otvos L. (2016). Polyvinyl alcohol nanofiber formulation of the designer antimicrobial peptide APO sterilizes Acinetobacter baumannii-infected skin wounds in mice. Amino Acids Vienna.

[131] Lee Y.J., Lee J.-H., Cho H.-J., Kim H.K., Yoon T.R., Shin H. (2013). Electrospun fibers immobilized with bone forming peptide-1 derived from BMP7 for guided bone regeneration. Biomaterials.

[132] Shao Z., Zhang X., Pi Y., Wang X., Jia Z., Zhu J., Dai L., Chen W., Yin L., Chen H. (2012). Polycaprolactone electrospun mesh conjugated with an MSC affinity peptide for MSC homing in vivo. Biomaterials.

[133] Foldvari M., Chen D.W., Nafissi N., Calderon D., Narsineni L., Rafiee A. (2016). Non-viral gene therapy: Gains and challenges of non-invasive administration methods. J. Control. Release.

[134] Penn M., Michler R.E., Espinal E., McGrath M.F., Firstenberg M.S., McCarthy P.M., Patel A.N. (2014). Stromal cell derived factor-1 over-expression immediately following surgical closure minimizes scar formation. J. Am. Coll. Surg..

[135] Ko J., Jun H., Chung H., Yoon C., Kim T., Kwon M., Lee S., Jung S., Kim M., Park J.H. (2011). Comparison of EGF with VEGF non-viral gene therapy for cutaneous wound healing of streptozotocin diabetic mice. Diabetes Metab. J..

[136] Dou C., Lay F., Ansari A.M., Rees D.J., Ahmed A.K., Kovbasnjuk O., Matsangos A.E., Du J., Hosseini S.M., Steenbergen C. (2014). Strengthening the skin with topical delivery of keratinocyte growth factor-1 using a novel DNA plasmid. Mol. Ther..

[137] Lee S., Jin G., Jang J.-H. (2014). Electrospun nanofibers as versatile interfaces for efficient gene delivery. J. Biol. Eng..

[138] Kim H.S., Yoo H.S. (2010). MMPs-responsive release of DNA from electrospun nanofibrous matrix for local gene therapy: In vitro and in vivo evaluation. J. Control. Release.

[139] Kim H.S., Yoo H.S. (2013). Matrix metalloproteinase-inspired suicidal treatments of diabetic ulcers with siRNA-decorated nanofibrous meshes. Gene Ther..

[140] Kim H.S., Yoo H.S. (2013). In vitro and in vivo epidermal growth factor gene therapy for diabetic ulcers with electrospun fibrous meshes. Acta Biomater..

[141] Luu Y.K., Kim K., Hsiao B.S., Chu B., Hadjiargyrou M. (2003). Development of a nanostructured DNA delivery scaffold via electrospinning of PLGA and PLA–PEG block copolymers. J. Control. Release.

[142] Saraf A., Baggett L.S., Raphael R.M., Kasper F.K., Mikos A.G. (2010). Regulated non-viral gene delivery from coaxial electrospun fiber mesh scaffolds. J. Control. Release.

[143] Yang Y., Xia T., Chen F., Wei W., Liu C., He S., Li X. (2012). Electrospun fibers with plasmid bFGF polyplex loadings promote skin wound healing in diabetic rats. Mol. Pharm..

[144] He S., Xia T., Wang H., Wei L., Luo X., Li X. (2012). Multiple release of polyplexes of plasmids VEGF and bFGF from electrospun fibrous scaffolds towards regeneration of mature blood vessels. Acta Biomater..

[145] Kataoka K., Harada A., Nagasaki Y. (2001). Block copolymer micelles for drug delivery: Design, characterization and biological significance. Adv. Drug Deliv. Rev..

[146] Kazunori K., Glenn S. K., Masayuki Y., Teruo O., Yasuhisa S. (1993). Block copolymer micelles as vehicles for drug delivery. J. Control. Release.

[147] Redhead H.M., Davis S.S., Illum L. (2001). Drug delivery in poly(lactide-co-glycolide) nanoparticles surface modified with poloxamer 407 and poloxamine 908: In vitro characterisation and in vivo evaluation. J. Control. Release.

[148] Thotakura N., Dadarwal M., Kumar R., Singh B., Sharma G., Kumar P., Katare O.P., Raza K. (2017). Chitosan-palmitic acid based polymeric micelles as promising carrier for circumventing pharmacokinetic and drug delivery concerns of tamoxifen. Int. J. Biol. Macromol..

[149] Wang Y., Ke X., Voo Z.X., Yap S.S.L., Yang C., Gao S., Liu S., Venkataraman S., Obuobi S.A.O., Khara J.S. (2016). Biodegradable functional polycarbonate micelles for controlled release of amphotericin B. Acta Biomater..

[150] Pan J., Liu N., Sun H., Xu F. (2014). Preparation and characterization of electrospun PLCL/poloxamer nanofibers and dextran/gelatin hydrogels for skin tissue engineering. PLoS ONE.

[151] Gong C., Wu Q., Wang Y., Zhang D., Luo F., Zhao X., Wei Y., Qian Z. (2013). A biodegradable hydrogel system containing curcumin encapsulated in micelles for cutaneous wound healing. Biomaterials.

[152] Kim J.H., Ramasamy T., Tran T.H., Choi J.Y., Cho H.J., Yong C.S., Kim J.O. (2014). Polyelectrolyte complex micelles by self-assembly of polypeptide-based triblock copolymer for doxorubicin delivery. Asian J. Pharm. Sci..

[153] Oyarzun-Ampuero F., Vidal A., Concha M., Morales J., Orellana S., Moreno-Villoslada I. (2015). Nanoparticles for the Treatment of Wounds. Curr. Pharm. Des..

[154] Kalwar K., Sun W.-X., Li D.-L., Zhang X.-J., Shan D. (2016). Coaxial electrospinning of polycaprolactone@chitosan: Characterization and silver nanoparticles incorporation for antibacterial activity. React. Funct. Polym..

[155] Chen C.-H., Chen S.-H., Shalumon K.T., Chen J.-P. (2015). Dual functional core–sheath electrospun hyaluronic acid/polycaprolactone nanofibrous membranes embedded with silver nanoparticles for prevention of peritendinous adhesion. Acta Biomater..

[156] Ghosh Auddy R., Abdullah M.F., Das S., Roy P., Datta S., Mukherjee A. (2013). New guar biopolymer silver nanocomposites for wound healing applications. BioMed Res. Int..

[157] Hazer D.B., Hazer B., Dinçer N. (2011). Soft tissue response to the presence of polypropylene-*g*-poly(ethylene glycol) comb-type graft copolymers containing gold nanoparticles. J. Biomed. Biotechnol..

[158] Leu J.-G., Chen S.-A., Chen H.-M., Wu W.-M., Hung C.-F., Yao Y.-D., Tu C.-S., Liang Y.-J. (2012). The effects of gold nanoparticles in wound healing with antioxidant epigallocatechin gallate and α-lipoic acid. Nanomed. Nanotechnol. Biol. Med..

[159] Raguvaran R., Manuja B.K., Chopra M., Thakur R., Anand T., Kalia A., Manuja A. (2017). Sodium alginate and gum acacia hydrogels of ZnO nanoparticles show wound healing effect on fibroblast cells. Int. J. Biol. Macromol..

[160] Martinez L.R., Han G., Chacko M., Mihu M.R., Jacobson M., Gialanella P., Friedman A.J., Nosanchuk J.D., Friedman J.M. (2009). Antimicrobial and healing efficacy of sustained release nitric oxide nanoparticles against *Staphylococcus aureus* skin infection. J. Investig. Dermatol..

[161] Rather H.A., Thakore R., Singh R., Jhala D., Singh S., Vasita R. (2017). Antioxidative study of cerium oxide nanoparticle functionalised PCL-Gelatin electrospun fibers for wound healing application. Bioact. Mater..

[162] Quignard S., Coradin T., Powell J.J., Jugdaohsingh R. (2017). Silica nanoparticles as sources of silicic acid favoring wound healing in vitro. Colloids Surf. B Biointerfaces.

[163] Wu H., Li F., Wang S., Lu J., Li J., Du Y., Sun X., Chen X., Gao J., Ling D. (2018). Ceria nanocrystals decorated mesoporous silica nanoparticle based ROS-Scavenging tissue adhesive for highly efficient regenerative wound healing. Biomaterials.

[164] Wang C., Hou W., Guo X., Li J., Hu T., Qiu M., Liu S., Mo X., Liu X. (2017). Two-phase electrospinning to incorporate growth factors loaded chitosan nanoparticles into electrospun fibrous scaffolds for bioactivity retention and cartilage regeneration. Mater. Sci. Eng. C.

[165] Gutha Y., Pathak J.L., Zhang W., Zhang Y., Jiao X. (2017). Antibacterial and wound healing properties of chitosan/poly(vinyl alcohol)/zinc oxide beads (CS/PVA/ZnO). Int. J. Biol. Macromol..

[166] Sanad R.A.-B., Abdel-Bar H.M. (2017). Chitosan–hyaluronic acid composite sponge scaffold enriched with Andrographolide-loaded lipid nanoparticles for enhanced wound healing. Carbohydr. Polym..

[167] Blakney A., Jiang Y., Woodrow K., Krogstad E. (2014). Delivery of multipurpose prevention drug combinations from electrospun nanofibers using composite microarchitectures. Int. J. Nanomed..

[168] Krogstad E.A., Woodrow K.A. (2014). Manufacturing scale-up of electrospun poly(vinyl alcohol) fibers containing tenofovir for vaginal drug delivery. Int. J. Pharm..

